# Length Dependent Folding Kinetics of Alanine-Based Helical Peptides from Optimal Dimensionality Reduction

**DOI:** 10.3390/life11050385

**Published:** 2021-04-24

**Authors:** Krzysztof Kuczera, Robert Szoszkiewicz, Jinyan He, Gouri S. Jas

**Affiliations:** 1 Department of Chemistry, The University of Kansas, Lawrence, KS 66045, USA; j237h128@ku.edu; 2 Department of Molecular Biosciences, The University of Kansas, Lawrence, KS 66045, USA; 3 Biological, and Chemical Research Centre, Faculty of Chemistry, University of Warsaw, Żwirki i Wigury 101, 02-089 Warsaw, Poland; rszoszkiewicz@chem.uw.edu.pl; 4 Department of Pharmaceutical Chemistry, The University of Kansas, Lawrence, KS 66047, USA; gouri.jas@ku.edu

**Keywords:** helix folding, molecular dynamics simulation, optimal dimensionality reduction, kinetic models

## Abstract

We present a computer simulation study of helix folding in alanine homopeptides (ALA)n of length n = 5, 8, 15, and 21 residues. Based on multi-microsecond molecular dynamics simulations at room temperature, we found helix populations and relaxation times increasing from about 6% and ~2 ns for ALA5 to about 60% and ~500 ns for ALA21, and folding free energies decreasing linearly with the increasing number of residues. The helix folding was analyzed with the Optimal Dimensionality Reduction method, yielding coarse-grained kinetic models that provided a detailed representation of the folding process. The shorter peptides, ALA5 and ALA8, tended to convert directly from coil to helix, while ALA15 and ALA21 traveled through several intermediates. Coarse-grained aggregate states representing the helix, coil, and intermediates were heterogeneous, encompassing multiple peptide conformations. The folding involved multiple pathways and interesting intermediate states were present on the folding paths, with partially formed helices, turns, and compact coils. Statistically, helix initiation was favored at both termini, and the helix was most stable in the central region. Importantly, we found the presence of underlying universal local dynamics in helical peptides with correlated transitions for neighboring hydrogen bonds. Overall, the structural and dynamical parameters extracted from the trajectories are in good agreement with experimental observables, providing microscopic insights into the complex helix folding kinetics.

## 1. Introduction

Because helices are crucial building blocks of protein and peptide structures, the details of their folding are of significant interest. The understanding of folding of model peptides is important for both the fundamental explanation of protein behavior as well as for explaining functions of biologically active peptides.

Helix folding has been the topic of numerous experimental and modeling studies, revealing many essential features of the process. Experimentally observed time scales associated with folding model helices are several hundred nanoseconds [1,2,3,4,5,6]. Uniform helix initiation along the peptide chain and an elongation time scale of 50 ns has been measured [7]. It was proposed more recently that the peptide helices form preferentially from the N- towards the C-terminus [8,9]. For the smallest and fastest folding helical pentapeptide a timescale of 10 ns was detected [10,11].

Computational modeling was able to reproduce measured helix content and relaxation times and provided a microscopic picture of helix folding (see [12]). Recent work includes applications of Milestoning [13,14] and Markov State Modeling [8]. We have performed experimental and computational studies for alanine-based peptides of varying lengths, determining helix content, relaxation kinetics, and folding pathways [2,13,15,16,17,18,19]. Helix content and folding rates varied under different force fields for the shorter peptides, and folding from the center was the dominant mechanism for the more extended system. More recently, we have studied the effects of pH variation on the stability and associated free energy landscape of the model peptide WH21 [20,21].

We present a molecular dynamics (MD) simulation study of length-dependent helix folding in model alanine homopeptides. These systems present homogenous models, employing the amino acid with the highest helical propensity [22,23,24,25,26] without significant sidechain complications. Analysis of their folding paths may provide a reference point for understanding the behavior of peptides of other sequences. We focused on four blocked alanine homopeptides of varying length, with 5, 8, 15 and 21 residues, further denoted as ALA5, ALA8, ALA15 and ALA21, respectively. ALA5 is chosen to model the shortest helix-forming peptide, WH5, for which structural and kinetic experiments have been performed [11]. ALA15 and ALA21 were used as models for previously studied helix-forming peptides of moderate size [12], while ALA8 is a representative of intermediate length peptides. We generated multi-microsecond MD trajectories at 300 K for these systems and extracted structural and dynamical information. Our MD results agreed qualitatively with a wide range of experimental data, providing microscopic insights into the helix folding mechanism. We found helix populations increasing from ~6% to ~60% for ALA5 to ALA21, and folding free energies decreasing linearly with the number of residues. The global relaxation times for helix folding increased exponentially with peptide length, from ~2 ns to ~500 ns in ALA5 to ALA21.

Helix folding trajectories were analyzed applying the optimal dimensionality reduction method, yielding coarse-grained kinetic models that gave a detailed representation of the folding process. We found that the shorter peptides, ALA5 and ALA8, tended to convert directly from coil to helix, while the longer peptides courses went through several intermediates. The coarse-grained aggregate states describing the helix, coil, and intermediates were heterogeneous, involving multiple peptide conformations. Folding occurred along multiple paths, and the relaxation was nonexponential. Interesting intermediate states were discovered on the folding paths, involving partially formed helices, turns, and compact coils. Statistically, helix initiation was favored at both termini, and the helix was most stable in the central region. An exciting finding was the presence of underlying universal local dynamics in helical peptides, involving correlated transitions of neighboring hydrogen bonds. Overall, this systematic study of alanine helix folding varying peptide length uncovered several exciting details about the formation process.

## 2. Methods

The simulated peptides were Ac-Ala_5_-NH_2_ (ALA5), Ac-Ala_8_-NH_2_ (ALA8), Ac-Ala_15_-NH_2_ (ALA15), and Ac-Ala_21_-NH_2_ (ALA21). The initial coordinates were generated in the helical and the extended conformations with CHARMM [27]. Peptides were solvated with TIP3P water and Na^+^ and Cl^−^ ions at 0.15 M concentration, followed by energy minimization, brief equilibration with harmonic restraints, and a ten ns equilibration under NPT conditions at 1 bar and 300 K. This assured a relaxed structure of peptide and solvent as well as appropriate box size. At this point the simulation systems started with the extended peptide structures were rather large (e.g., a cube of edge 7.6 nm with 13,593 water molecules for ALA15). For these systems, the equilibrated peptide coordinates were extracted from the trajectory and re-solvated and re-equilibrated at NPT. As a result, the final box sizes for these trajectories were comparable to those for systems started with the helical conformations. The system sizes and compositions are presented in the Supplemental Information. Finally, two molecular dynamics trajectories were generated for each peptide, starting from the helical (trajectory h) and extended (trajectory e) structures, each of 5 μs length for ALA5, 10 μs for ALA 8, 10 μs for ALA15, and 20 μs for ALA21. The MD simulations were performed with GROMACS 5.1.4 [28,29] using the CHARMM36m force field [30] and TIP3P water model [31]. A time step of 2 fs was employed, with NVT conditions employed for simplicity and efficiency, with the temperature of 300 K maintained by velocity scaling. Nonbonded cutoffs were 1.2 nm, and the PME method [32] was used to account for long-range electrostatic interactions.

Alpha helical contents were estimated by applying two methods. The first, further denoted as HB, counted the fraction of formed helical hydrogen bonds between the peptide C=O of residue i and the peptide NH of residue i + 4. Hydrogen bonds were considered to be present at an O…N distance below 3.6 Å. Our blocked peptides had maximum numbers of helical hydrogen bonds of 3, 6, 13, and 19 for ALA5, ALA8, ALA15, and ALA21, respectively. The second method, denoted by PP, was based on the fraction of residues with helical backbone conformation. Here, a residue was considered in the helical region of the Ramachandran map if its backbone dihedral angles (PP) were within 20° of the ideal helix conformation, (φ,ψ) = (−62°,−41°). In the blocked peptides, the maximum number of helical residues was 5, 8, 15, and 21.

Relaxation times associated with the MD trajectories’ folding dynamics were calculated from the autocorrelation functions (ACFs) of a range of global variables, including the radius of gyration and RMSD from helix, surface area, and number of hydrogen bonds. To probe local dynamics, we also calculated ACFs of length fluctuations of individual hydrogen bonds. The ACFs were fitted to two-exponential decays, as described in more detail in the Supplementary Materials.

Kinetic models were constructed analyzing MD trajectories by clustering, trajectory discretization, and kinetic coarse-graining, as described elsewhere ([17] also see Supplementary Materials for details). Briefly, discrete microstates are defined with CA atom RMSD clustering. Transition and kinetic matrices are based on transitions between microstate cores, with core radii chosen to match the slowest kinetic relaxation time to the times extracted directly from MD. Kinetic coarse-graining is carried out with PCCA+ [33], and effective rates in the low-dimensional spaces are determined with the optimal dimensionality reduction (ODR) method [34].

## 3. Results and Discussion

We used the multi-microsecond MD trajectories to characterize the studied peptides’ structures and dynamics, as described below. Unless otherwise specified, the results are averaged over the two independent trajectories for each system.

**Figure 1:**
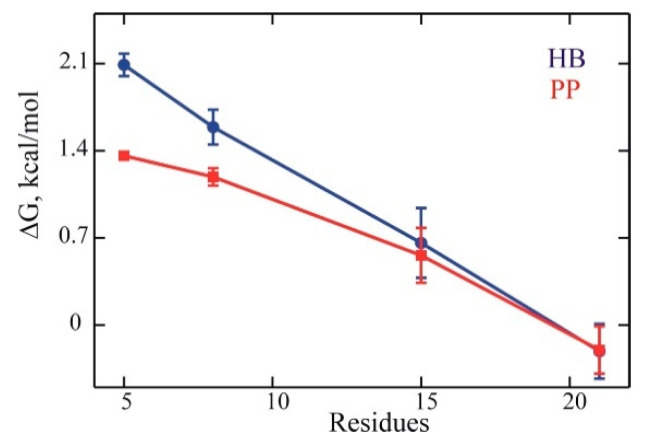
The variation of the folding free energy ΔG with peptide length. Blue: helix content measured by hydrogen bond count (HB). Red: helix content measured by backbone conformation (PP). Error bars show 95% confidence intervals.

**Helix content.** The details of helix content are presented in the Supplementary Materials. Generally, the fraction of α-helix content increased with peptide length, from 3–9% in ALA5 to about 60% in ALA21. The helicity measurements with the number of hydrogen bonds (HB) and backbone conformations (PP) were similar, especially in the longer peptides ALA15 and ALA21. The results of DSSP analysis of trajectory structures also agreed with the HB and PP measures. Figure 1 shows the folding free energy ΔG

(1) $$\Delta G=-RT\;ln\frac{f}{1-f}$$

as a function of the number of residues, where f is the fraction helix, R is the gas constant, and T is the temperature (T = 300 K). The plot’s slope is −0.1 to −0.2 kcal/mol per residue, showing a systematic but weak trend of increased stability of longer helices. This finding agrees with the experimental estimate for alanine-based peptides of −0.24±0.15 kcal/mol per residue at 300 K (see Comparison with experiment section).

**Figure 2:**
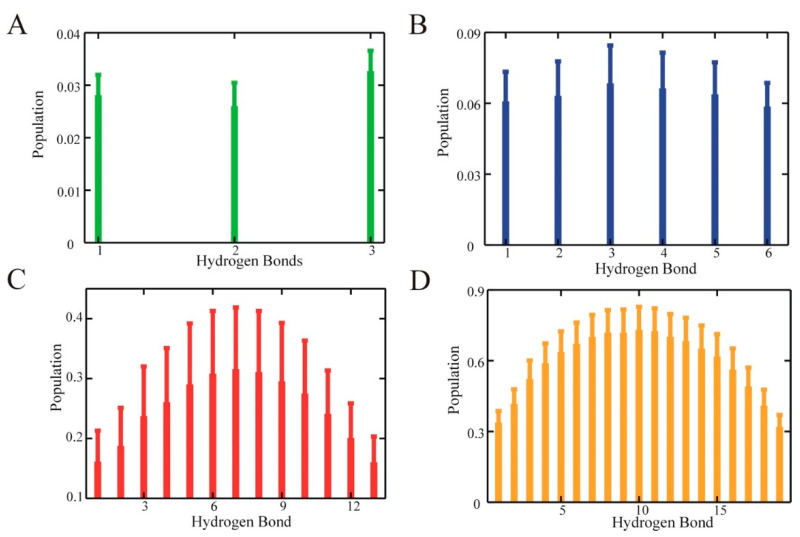
Average trajectory populations of individual helical hydrogen bonds. (**A**) ALA5. (**B**) ALA8. (**C**) ALA15. (**D**) ALA21. Generally, hydrogen bond i is between the C=O of residue i and NH of residue i + 4. The first hydrogen bond is between the C=O of the acetyl blocking group and NH of residue 4, the last is between the between the C=O of residue n − 3 and the amide blocking group NH_2_, with n = 5, 8, 15, 21. Error bars show 95% confidence intervals.

**Helical hydrogen bond populations.** The individual α-helical hydrogen bond populations are shown in Figure 2. Even in our long simulations, this data was noisy, though some interesting trends emerged. For the larger systems, ALA15 and ALA21, there was a clear tendency for the helix center’s highest population. This population was in accord with the results of previous simulations and experimental data (see Comparison with experiment section). For the smaller systems, evidence of interesting irregularities were present. Thus, in ALA5, which essentially formed a single helical nucleus, the terminal hydrogen bonds tended to have higher populations than the central one. In ALA8, the h-bond population distribution appeared essentially flat. Thus, the helix center’s enhanced stability appeared to be a feature of the longer helices.

**Conformations explored**. The three-dimensional structural conformations sampled in the simulations are discussed in more detail in the kinetic modeling section. A more general analysis of explored conformations is in the Supplementary Materials. In summary, ALA5 and ALA8 peptide simulations appeared to be mostly converged, with the two independent trajectories exploring very similar conformational space. In contrast, for ALA15 and ALA21, we found only partial structural overlap among the individual trajectories, suggesting that we had sampled only a portion of the available conformational space, especially for ALA21.

**Figure 3:**
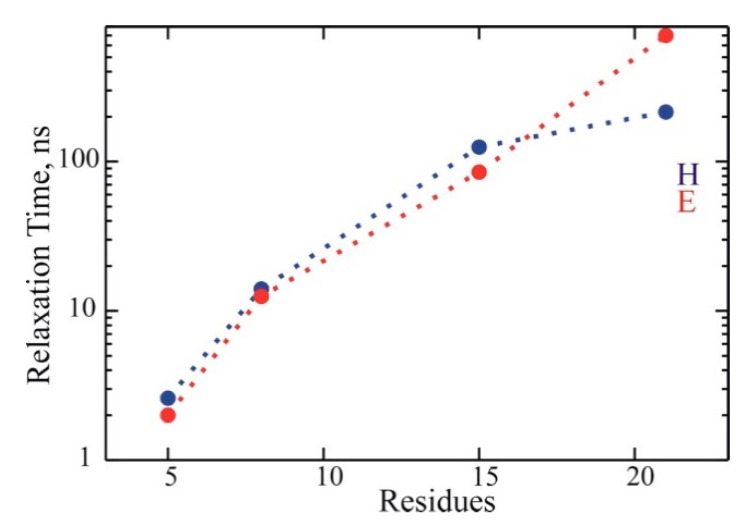
Slowest relaxation times from the MD trajectories of the blocked ALAn peptides, with n = 5, 8, 15 and 21. Blue: trajectories started from helical structure (H). Red: trajectories started from extended structure (E). Logarithmic scale.

**Dynamical timescales.** The timescales associated with the MD trajectories’ conformational dynamics were analyzed from the ACFs of local and global variables. The details are in the Supplementary Materials. A summary in Figure 3 shows a roughly exponential increase of global relaxation time with helix length. The slowest relaxation time was ~2 ns in ALA5, ~12 ns in ALA8, ~100 ns in ALA15, and ~500 ns in ALA21. For ALA5, ALA8, and ALA15, the two independent trajectories’ relaxation times were quite similar. It was strikingly different for ALA21, where the slowest relaxation time was ~200 ns in trajectory h and about 700 ns in trajectory e, indicating that the ALA21 simulations were not converged. These long time scales were similar across many variables for each system, including the radius of gyration, RMSD from helix, and helix content measures; we assign this relaxation time to global helix folding.

From the two exponential data fits to the ACFs (shown in the Supplementary Materials), we can identify a shorter relaxation time scale, which is ~0.5 ns in ALA5, ~1 ns in ALA8, ~2 ns in ALA15, and ~20 ns (3–40 ns range) in ALA21. Remarkably, the faster time increased linearly with the increase in peptide length. As explained, this appeared to be a process involving local hydrogen bond dynamics, and more detail is in the local vs. global dynamics section.

**Figure 4:**
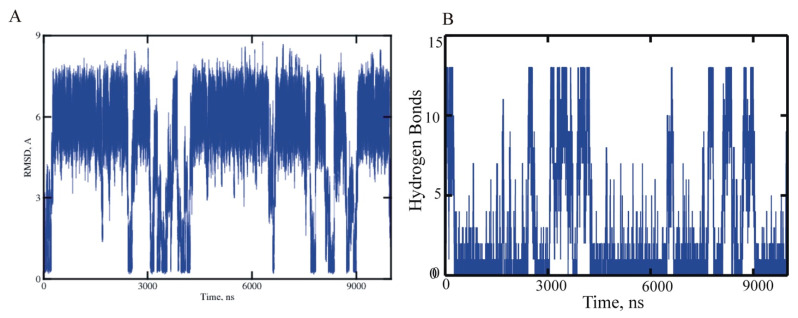
ALA15 MD trajectory h, 10 microseconds at 300 K exhibits multiple helix folding and unfolding events. (**A**) CA atom RMSD from ideal helix. (**B**) Number of formed alpha-helical hydrogen bonds. Data sampled every 100 ps.

**Figure 5:**
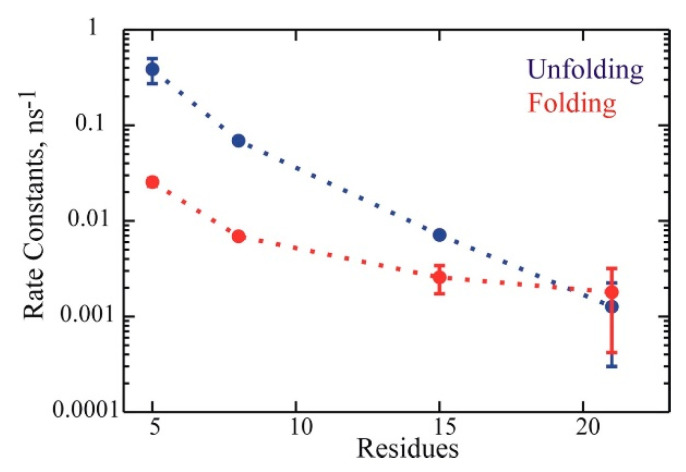
Folding rates k_f_ (red) and unfolding rates k_u_ (blue) for the peptide series ALAn, n = 5, 8, 15, 21. Rate constants calculated from MD trajectories, in units of ns^−1^.

**Global helix folding and unfolding.** There were multiple helix folding and unfolding events that occurred in the MD trajectories. Examples representing these events for ALA15 are in Figure 4. Results for the rest of the peptides are in the Supplementary Materials. As presented in the Supplementary Materials, the calculated helix fractions and global relaxation times were used to estimate the folding rate k_f_ and the unfolding rate k_u_ as a function of length in the helical peptide series. The folding and unfolding rates for the four peptides exhibited a systematic tendency to decrease with peptide length, shown in Figure 5. As expected from the helix populations, unfolding was faster for the shorter peptides, while folding was faster for ALA21. The rates for ALA21 were comparable to those found for the WH21 peptide, which has a similar length and a slightly diverse amino acid composition [17]. The rates for ALA5 were comparable to measurements for the helical pentapeptide WH5 [11,18].

**Kinetic models**

**Figure 6:**
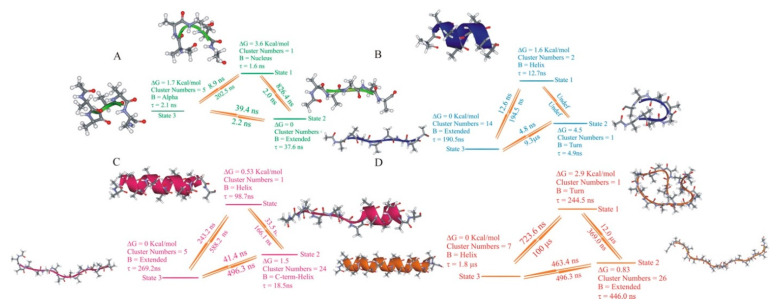
Coarse-grained kinetic models of dimension N = 3 for the ALAn peptides. (**A**) ALA5 model based on discretization with N_c_ = 30 clusters. (**B**) ALA8 model based on N_c_ = 17 clusters. (**C**) ALA15 model based on N_c_ = 45 clusters. (**D**) ALA21 model based on N_c_ = 34. For each aggregate set the representative structure is shown, i.e., the central structure of the most populated cluster among all those grouped together in the given set.

We generated multiple folding kinetic scenarios applying optimal dimensionality reduction (ODR) for the four studied alanine homopeptides. First, we performed clustering with four different cluster radii for each peptide, which led to sets of clusters with varying resolution: N_c_ = 5–62 clusters for ALA5, N_c_ = 8–305 ALA8, N_c_ = 11–491 for ALA15, and N_c_ = 34–605 for ALA21. The cluster center conformations are denoted as microstates. Next, trajectory discretizations were performed for each clustering scheme, assigning each trajectory frame to a cluster/microstate. Finally, the ODR procedure was applied to create low-dimensional coarse-grained models with N = 2–5 aggregate states. Details of the procedure and outcomes are in the Supplementary Materials. The summary of the kinetic models with N = 3 states are in Figure 6.

**Assigning helix and coil States.** Here we followed a general scheme for assigning aggregate sets to the structure types. We assigned the helix set as the lowest CA atom RMSD from the ideal helix and the largest number of helical hydrogen bonds, with helix content confirmed by molecular graphics analysis. We assigned the coil/unfolded set as the one made up of the largest number of clusters, high RMSD from helix, and with the presence of extended/PPII peptide conformers confirmed by molecular graphics. Any remaining sets were classified as folding intermediates.

**Figure 7:**
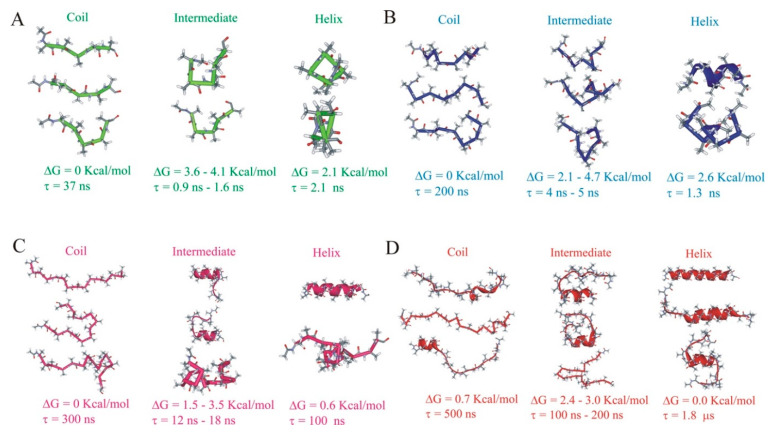
Summary of properties of helix, coil and intermediate aggregate states in the ORD models at multiple resolutions (**A**) ALA5. (**B**) ALA 8. (**C**) ALA15. (**D**) ALA21.To illustrate the inhomogeneous nature of the coarse-grained aggregate sets, central structures of several clusters grouped in a given set are shown, together with their properties. ΔG is the free energy relative to the most stable form (coil for ALA5-ALA15, helix for ALA21). The lifetime is τ = −1/R_ii_, with **R** the reduced rate matrix and i the set number.

In many cases, the aggregate sets consisted of a large number of raw clusters/microstates. For ease of understanding, we illustrated their structural properties by visualizing a single representative structure—the central structure of the most populated cluster within the set (structures in Figure 6). An expanded view of the crucial structures is in Figure 7.

**ODR relaxation times.** Mostly, we found that the two fastest relaxation times of the reduced-dimensional rate matrix R, given in Table 1, well reproduced the corresponding times found in the full kinetic matrix K (presented in Tables S11–S14, Supplementary Materials), with typical deviations of 10–30%. The exceptions were the highest resolution models for ALA21 (N_c_ = 194 and 605), for which deviations were much more prominent. The trend was for both the relaxation times to increase with helix length. The slowest ODR timescales corresponded to helix-coil transitions and were set to match the most extended MD time scales from the microstate core radius R_c_ choice. The second-slowest time scales were model predictions and corresponded to transitions between helix, coil, and intermediate states, shown in Figure 6.

**Two-state models (N = 2)**. The summary of the lowest level, the two-state model, is given in Table 2. These models were in good accord with the results extracted directly from the MD trajectories (see Figure 4 above and Supplementary Materials). The rate constants typically fell within 50% of the MD values, while the free energies were mostly within 0.5 kcal/mol. The exceptions were again the highest resolution models for ALA21, with N_c_ = 194 and 605, which exhibited more significant deviations, predicting unfolding rates of about 1$\times 10^{-5}{\;\mathrm{ns}}^{-1}$ and ΔG = −4 kcal/mol. Thus, most of our two-state models captured the system structure and dynamics’ main features, with the additional insight of partition of the microstates into the helix and coil aggregate sets. The most noteworthy feature was the heterogeneity of the sets. The helix set typically consisted of a fully helical structure and several partly folded forms. The coil set included the majority of the microstates, including extended, polyproline (PPII), turn, beta, and some partially folded helices.

The properties of the coarse-grained models of the four peptides with N = 3–5 aggregate states are shown below. Kinetic schemes and representative structures for the N = 3 models are in Figure 6. Figure 7 presents a summary of the structures sampled in the helix, coil, and intermediate aggregate sets at different resolution levels, illustrating the inhomogeneous nature of the aggregate sets.

**ALA5.** Here the unfolded, or coil state was the most highly populated, and the helix was a minor conformer (~3–9% population, as in above). The properties of the N = 3 model for N_c_ = 30 are presented in Figure 6A. At the lowest resolution, i.e., for the lowest numbers of microstates, N_c_ = 5 and 9, the helix set representative structures were partial helices. At the higher resolutions, N_c_ = 30 and 62, we found ideal helix structures as representatives (Figure 7A). Other structures included in the helix set included partially folded forms with single helical h-bonds (ACEO…HN4, 1CO…HN5, 2CO…NT) and 3_10_ helical turns. Intermediates on the folding pathway included a compact folding nucleus with bifurcated hydrogen bonds between the ACE CO group and HN3 and HN5 and turns exhibiting no hydrogen bonding. The intermediates had lifetimes of 1–2 ns, comparable to the helix, high free energies, about 4 kcal/mol above the coil state, and more than 2 kcal/mol above the helix. The rates of formation of the intermediates from both helix and coil were relatively slow so that the direct helix−coil transition should have dominated here. The unfolded set combined several extended and PPII type conformers and various turns with internal hydrogen bonds (Figure 7A).

**ALA8.** Here the helix was also a minor conformer (7–12% population, see above), though with a more significant contribution than in ALA5. The ALA8 N = 3 model for N_c_ = 17 was presented in Figure 6B. For ALA8, we found fully helical representative structures in all of the explored levels of resolution N_c_. The helical set included various partly folded helices with a majority of hydrogen bonds formed. The intermediates represented a nascent beta-hairpin (with 3CO…HN6 and 3NH…OC6 hydrogen bonds), other hydrogen-bonded turns, and helices with up to one half-formed h-bonds (Figure 7B). The intermediates had lifetimes of 4–5 ns, significantly shorter than the helix or coil. The intermediate free energies were about 2–5 kcal/mol above the coil and 1–3 kcal/mol above the helix. As in ALA5, the intermediates’ formation rates were relatively low, and the direct helix−coil transition should be the dominant process. The unfolded forms included mostly extended, PPII, and turn populations of structures (Figure 7B).

**ALA15.** Here the helix was the crucial conformation, with 25–28% population in the MD. The N = 3 model for N_c_ = 45 is presented in Figure 6C. For ALA15 at the lowest resolution, N_c_ = 11, the helical set consisted of the ideal helix only. At higher resolutions, it also included partially folded helices (Figure 7C). The intermediates included partial helices at the N- and C-termini and a compact coil formed by turns with four hydrogen bonds. The intermediate lifetimes were 12–18 ns, and their free energies were about 1.5–3.0 kcal/mol above the coil. In ALA8, the rates of intermediate formation were comparable to the helix-coil transition rates. Thus, for this peptide, one might expect multiple competing folding pathways between helix and coil sets. The coil set included mostly extended and PPII structures, various turns, and compact unstructured populations (Figure 7C).

**ALA21.** In this peptide system, the major conformer was the helix, with approximately 60% population. The N = 3 model for N_c_ = 34 is presented in Figure 6D. For ALA21, the helical set was heterogeneous in all models. Due to the higher resolution models’ inconsistencies, for ALA21, only the N_c_ = 34 and N_c_ = 76 models are analyzed here, with the remaining data placed in the Supplementary Materials. The helical set included the complete helix and partial helices, involving both one and two helical sections (Figure 7D). The intermediates included a helix-turn-helix motif, C-terminal helices, turns, and compact coils. The intermediate lifetimes were about 100–200 ns, much shorter than the helix or coil. The free energies of the intermediate states were about 2–3 kcal/mol above the helix. The intermediates’ formation rates were relatively slow, but their large numbers in this long peptide indicated that multiple folding pathways between helix and coil sets should be present in ALA21. The unfolded/coil set included extended/PPII structures, helical nuclei at N- and C-termini, and compact coil states (Figure 7D).

**Measures of aggregate set inhomogeneity.** The coarse-grained aggregate sets in our models were determined at the kinetic level, by analysis of the sign structure of the eigenvectors of the transition matrix [33]. To analyze the structural inhomogeneity of these sets, we calculated average CA RMSD for pairs of clusters within each set and between sets. Detailed definitions and results for selected models are presented in the Supplementary Information. At higher resolutions, the within-set distances in the helix sets were about 1.0–1.6 Å in ALA5, 1.4–1.7 Å in ALA8, 4–5 Å in ALA15 and 7–8 Å in ALA21. For comparison, within-set distances in the coil sets were about 1.8–2.0 Å in ALA5, 2.7–3.8 Å in ALA8, 6–7 Å in ALA15 and 9 Å in ALA21. Within-set averages for intermediates were estimated at 1.6–2.0 Å in ALA8, 4–6 Å in ALA15 and 7–10 Å in ALA21. Overall, the inhomogeneity was relatively lowest for the helices, although absolute values found for the longer peptides were quite large. Inhomogeneities exhibited similar ranges for the coils and intermediates, and between-set distances were comparable to within-set values for coils.

**Figure 8:**
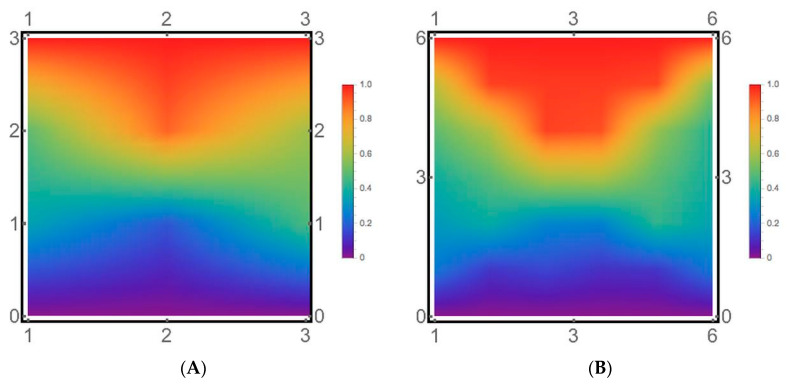

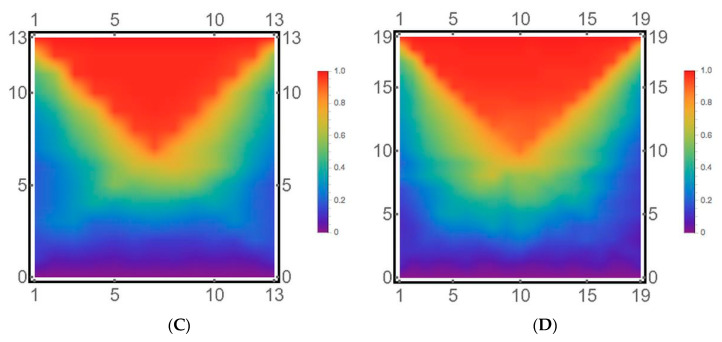
Statistical picture of helix folding. Shown are heat maps of conformer populations P(HBi,NHB). Columns correspond to individual hydrogen bond numbers HBi, i = 1, 2,…, n−2, with n–the total residue numbers. Data in each row are obtained by averaging over a subset of structures with a defined total value of formed hydrogen bonds NHB. The index of the first residue labels the individual hydrogen bonds, i.e., HB1 is the hydrogen bond between the acetyl group C=O and the amide N-H group of ALA4 (ACE-C=O…HN4), HB2 involves 1C=O…HN5, and the last hydrogen bond involves (n−3) C=O…H_2_NT, with NT being the C-terminal amide blocking group nitrogen. The color represents the value of the population: blue is lowest, green is intermediate, red is highest. (**A**) ALA5. (**B**) ALA8. (**C**) ALA15. (**D**) ALA21.

**Helix folding pathways.** To further characterize these four peptides’ folding pathways, we combined the kinetic network analysis with the MD data’s statistical analysis in Figure 8 (raw data is in Supplementary Materials). Figure 8 shows a heat map P (HBi, NHB) of the populations of individual helical hydrogen bonds HBi, i = 1, 2,…, n−2, as a function of the number of total helical hydrogen bonds present NHB (n is the number of residues in the peptide). These maps showed significant similarities in the folding statistics.

The propensity for helix initiation, or formation of the first helical hydrogen bond, may be evident in the NHB = 1 data in Figure 8. In ALA5, forming the two-terminal bonds occurred first, with the C-terminal end favored most strongly, followed by the central hydrogen bond addition. In ALA8−ALA21, a preference was first formed in the two-terminal bonds, a primarily uniform propagation to the NB = 3 nucleus, followed by preferential helix propagation from the center to the termini. There was a trend of the highest hydrogen bond population in the helix center, more pronounced in the longer peptides. For ALA21, the three central hydrogen bond populations reached about 90% in the NHB = 11 slice, with helix content decreased to 20–30% at the termini. With NHB = 15, the eleven central h-bonds of ALA21 had populations above 95%.

Overall, the statistical tendency was for preferred initiation at the termini, mostly uniform nucleation and propagation from the center toward both ends for the helix lengths studied here. As can be seen from the intermediate structure analysis above, in the longer peptides, this picture is due to averaging of helix fragments in the center, both terminal regions, and helix-turn-helix motifs.

**Transition states.** Transition states were calculated from the transition path analysis tool of Emma1.4 [35]. For ALA15, structures with committor values close to q = 0.5 corresponded to partial helices at the N- and C-termini (Figure 7C, intermediates). For ALA21, the coarse-grained sets close to a transition state included partially folded states with central helical regions and an interesting intermediate with partial 3_10_ helical structure (Figure 7D, intermediates). In ALA8, the structures closest to the TS (q = 0.69) exhibited partial helical structure (Figure 7B, intermediates). In the shortest system of our studied peptides, ALA5, the TS-like states were not resolved.

**Figure 9:**
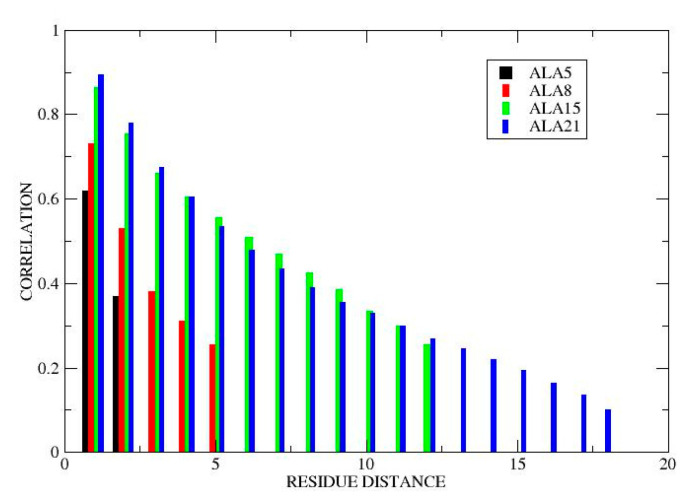
Correlated fluctuations of neighboring hydrogen bonds. Plot shows correlation coefficients between length fluctuations of nearest neighbor (i…i + 1), next-nearest neighbor (i…i + 2), etc. hydrogen bonds, averaged over the peptide chain. Data points are shifted slightly along the x axis to avoid overlap. Black: ALA5, red: ALA8, green: ALA15, blue: ALA21.

**Local vs. global MD.** Since hydrogen bonds are the basic helical structure units, we have also calculated the average relaxation times of individual hydrogen bonds in the four peptides (details in SI). Most individual h-bond ACFs could be well represented as double exponentials, with the longer relaxation times approximately equal to the global times found for RMSD and other global variables (see above). The faster individual hydrogen bond motions occurred on timescales of 100–200 ps in ALA5, 0.7–0.9 ns in ALA8, 1.5–1.8 ns for ALA15, and about 7 ns ALA21. These values roughly agree with the second-slowest relaxation times seen in the global variables (see Dynamical timescales section), indicating that such motions make essential contributions to peptide dynamics in solution. Strong correlations were found between fluctuations of neighboring hydrogen bonds, with correlation coefficients of up to 0.9 for nearest neighbors in ALA15 and ALA21, and 0.6–0.7 in ALA5 and ALA8 (Figure 9). These results suggest that the fundamental mechanism for conformational transitions of the helical polypeptide chain involves cooperative breaking/formation of blocks of several consecutive hydrogen bonds. Motions on a similar time scale have been observed experimentally [7,9,17].

**Comparison with experiment.** There are limited data for alanine homopeptides, but extensive studies with alanine-based model helices of similar size can be used for qualitative comparisons. The observed helix populations at room temperature are ~10% for ALA5 [18], ~20% for a related pentapeptide WH5 [10], and ~46% for the 21-residue WH21 system [17]. These are comparable to our computational estimates of 3–6% for ALA5 and 60% for ALA21.

To obtain an estimate of the slope of the folding free energy with the number of residues ΔG/Δn, we used published data for enthalpy ΔH/Δn=−0.9±0.1 kcal/mol per residue [36] and entropy ΔS/Δn=−2.2±0.4 cal/(mol K) per residue [37], to obtain ΔG/Δn=ΔH/Δn−TΔS/Δn=−0.24±0.15 kcal/mol per residue at T = 300 K. Our computed slope agreed with this estimate within the errors.

A global relaxation times of tens to hundreds of nanoseconds has been observed for alanine-based peptides of various lengths at room temperature, including ~10 ns for the WH5 pentapeptide [11] and 300 ns for WH21 [2,17]. These are in good agreement with our simulated values of ~2 ns for ALA5 and 500 ns for ALA21. The nonexponential nature of the helix folding was observed experimentally [16,17]. Faster relaxation components have also been experimentally detected in other peptides, e.g., at ~1 ns in WH5 [11] and ~20 ns in WH21 [16,17], in a similar range to our time scales of 200 ps for ALA5 and 3–40 ns for ALA21. A helix propagation rate of 65 ns was also recently determined [7], which roughly agrees with our faster ALA21 component.

In accord with our simulated hydrogen bond population patterns, a higher melting temperature and slower relaxation in the helix center were observed experimentally [3,38] (Figure 2).

Overall, the multiple simulated features were in reasonable agreement with observations made for related peptide systems. This suggests that our simulations, using the CHARMM36m protein force field and TIP3P water model, presented realistic representations of peptide folding for helices of various lengths. In recent years there has been increasing focus on analyzing the accuracy and reliability of computer simulation by comparison with experimental data. Studies include the prediction of secondary structures [39], folding [18,40,41] and the ability to describe unfolded states [42]. Based on these investigations, it appears that modern protein force fields are increasingly accurate in terms of major state populations and relaxation time scales. However, the microscopic details of folding pathways remain difficult to verify experimentally.

## 4. Conclusions

Here, we present the results of multi-microsecond molecular dynamics simulations of four blocked alanine peptides—ALA5, ALA8, ALA15, and ALA21—to analyze the structure and folding pathways of these helix forming systems as a function of length. A progressive increase of alpha-helix content for these peptides, from ~6% ALA5 to ~60% in ALA21, was observed, based on the trajectory analysis. The systems undergo multiple transitions between helix and coil, facilitating the determination of basic kinetic parameters such as the global relaxation time $\tau_{2}$, and helix folding and unfolding rates from MD simulations. An exponential increase in the folding relaxation times was found with growing peptide length, from ~2 ns in ALA5 to ~500 ns in ALA21. The folding and unfolding rates progressively decrease with the increase in the number of residues.

We generated coarse-grained kinetic models based on the ODR method to gain further insight into the folding mechanisms. In this model, we varied the numbers of the underlying microstates for trajectory discretization (number of clusters N_c_) and the number of coarse-grained states in the coarse-grained kinetic models (number of aggregate sets N). Combining the results at several resolutions, we characterize the peptide dynamics’ common features for the studied systems. In the lowest resolution two-state models (N = 2), the kinetic parameters were essentially the same as those extracted from the MD trajectories directly. From models with N = 3–5, we described transitions between the helix, coil, and intermediate states and the underlying peptide structures. Thus, the coarse-grained helix sets involved entire and partially folded helices, and the coils were mostly with extended, PPII, and turn conformations. The intermediates had the lowest populations and shortest lifetimes and included turns and partially formed helices, with details varying with peptide length.

Our models predict the dominance of a two-state process, helix−coil, in the ALA5 and ALA8. The formation of intermediates was well resolved along the folding pathway of ALA15 and ALA21 systems. A remarkable insight from these calculations was that both ‘helix’, ‘coil’, and ‘intermediate’ states were inhomogeneous, combining several microstates (i.e., clusters). This inhomogeneity was not surprising for the ‘coil’ state, as it involves a large ensemble of structures based on both experimental and computational studies. However, the presence of inhomogeneity in the ‘helix’ state is an exciting finding. This heterogeneity implies multiple helix folding pathways, even in two-state models or higher dimension models that involve helix−coil transitions.

Following the statistics of helix folding, our MD results indicate that these peptides initiate folding at the termini, and the formation of the helical nucleus with three hydrogen bonds occurs approximately uniformly along the chain. The helix is most stable in the center and propagates towards both termini. This simple picture hides the presence of partly folded structures at the N- and C-terminus and helix-turn-helix motifs, especially significant for the most extended ALA21 system.

Besides the slowest process, assigned to helix folding, our trajectories involve processes on a slower time scale. These rates increase roughly linearly with peptide size, ranging from single nanoseconds and lower in ALA5 and ALA8, to tens of nanoseconds in ALA15 and ALA21. These time scales are much faster than those involving helix−coil relaxations or transitions to folding intermediates, but they agree with the typical relaxation times of length fluctuations of individual hydrogen bonds. Additionally, fluctuations of neighboring hydrogen bonds are highly correlated. Here we propose that these faster dynamical processes, involving correlated breaking and forming blocks of several neighboring hydrogen bonds, are the fundamental mechanism of conformational transitions in helical peptides. These transitions would occur in all peptide conformations, folded, unfolded, and intermediate, and yield a strong signal in the observed dynamics.

Several features found in our simulations agree qualitatively with available data on model helix folding in alanine-based peptides, including the variation of helix content and global relaxation time with length, the presence of a faster relaxation component, and high stability of the helix center. This observation indicates that the CHARMM36m protein force field with TIP3P water can generate realistic helix formation models in an aqueous solution. In general, the details of helix folding will depend on the sequence, due to specific sidechain effects, as demonstrated in multiple studies (e.g., [20,43]). The analysis of alanine peptide folding presented here provides a baseline for understanding helix formation in other peptides. Our studies show the behavior of pure alanine systems, without the effects of complicating sidechain interactions. Thus, these results may be used to uncover specific sidechain effects in future studies on folding pathways of more heterogeneous systems.

It is interesting to compare the optimal dimensionality reduction (ODR) method to the alternative approaches for atomistic modeling of long-term kinetics—Milestoning and Markov State Modeling (MSM). MSM and Milestoning are more established and their theoretical backgrounds have been well described. The relative advantage of Milestoning is that it can be used to describe processes with arbitrarily long timescales, only requiring large numbers of short trajectories between milestones [44,45,46]. Its relative disadvantage is the technical difficulty of the method, which appears hard to automate. In both MSM and ODR the whole conformational space must be explored in several long trajectories, which limits these approaches to processes of moderate length [47]. The advantage of MSM is the availability of relatively easy to use tools [48,49]. The relative disadvantage is the difficulty in structural interpretation of MSM results. For ODR the advantages are the ability of using a relatively small number of microstates to discretize the conformational space and ease of structural interpretation. The disadvantages at this time are lack of automated tools and incomplete exploration of mathematical and physical properties of the method.

Overall, our studies revealed several new and exciting features of the microscopic mechanism of helix folding events. We found that it was necessary to expand the ‘helix’ concept to include partly folded structures. We uncovered exciting differences in the folding paths with peptide length—mostly direct transitions for the shorter ones and both direct and through intermediates for the longer ones. Additionally, the folding intermediates varied for peptides of different lengths. Importantly, our results imply the presence of underlying universal local dynamics in helical peptides, involving correlated transitions of neighboring hydrogen bonds.

## Figures and Tables

**Table 1:** Summary of the slowest relaxation times in ODR kinetic models. The ranges correspond to models with varying values of clusters N_c_ and aggregate states N described in SI. Units: ns.

	Longest	Second-Longest
ALA5	2.0–2.4	1.3–1.6
ALA8	12.6–13.4	3.2–5.8
ALA15	101–106	17–23
ALA21 *	370–460	200–240

* Using Nc = 34 and 76 only.

**Table 2:** Properties of two-state ODR models (N = 2). Rate constants for folding (k_f_) and unfolding (k_u_) are the R matrices’ off-diagonal elements. The folding free-energy ΔG is calculated from the populations of the aggregate states. The ranges of values come from models with different resolutions N_c_.

System	k_u_, ns^−1^	k_f_, ns^−1^	ΔG, kcal/mol
ALA5	$(3.9–4.7)\times 10^{-1}$	$(2.4–2.9)\times 10^{-2}$	1.6–1.7
ALA8	$(7.0–7.9)\times 10^{-2}$	$(3.6–7.8)\times 10^{-3}$	1.3–1.8
ALA15	$(6.6–9.2)\times 10^{-3}$	$(2.9–4.8)\times 10^{-3}$	0.4–0.5
ALA21 *	$(5.3–5.5)\times 10^{-4}$	$(1.7–2.1)\times 10^{-3}$	−0.7–−0.8

* Using Nc = 34 and 76 only.

## Data Availability

The data presented in this study are available on request from the corresponding author. The data are not publicly available due to large volume.

## Supplementary Materials

The following are available online at https://www.mdpi.com/article/10.3390/life11050385/s1, Additional details about the simulations and their analysis.

## References

[1] Williams S., Causgrove T.P., Gilmanshin R., Fang K.S., Callender R.H., Woodruff W.H., Dyer R.B. (1996). Fast events in protein folding: Helix melting and formation in a small peptide. Biochemistry.

[2] Thompson P.A., Munoz V., Jas G.S., Henry E.R., Eaton W.A., Hofrichter J. (2000). The helix-coil kinetics of a heteropeptide. J. Phys. Chem. B.

[3] Ianoul A., Mikhonin A., Lednev I.K., Asher S.A. (2002). UV resonance Raman study of the spatial dependence of alpha-helix unfolding. J. Phys. Chem. A.

[4] Thompson P.A., Eaton W.A., Hofrichter J. (1997). Laser temperature jump study of the helix reversible arrow coil kinetics of an alanine peptide interpreted with a ‘kinetic zipper’ model. Biochemistry.

[5] Jas G.S., Eaton W.A., Hofrichter J. (2001). Effect of viscosity on the kinetics of alpha-helix and beta-hairpin formation. J. Phys. Chem. B.

[6] Eaton W.A., Munoz V., Hagen S.J., Jas G.S., Lapidus L.J., Henry E.R., Hofrichter J. (2000). Fast kinetics and mechanisms in protein folding. Annu. Rev. Biophys. Biomol. Struct..

[7] Fierz B., Reiner A., Kiefhaber T. (2009). Local conformational dynamics in alpha-helices measured by fast triplet transfer. Proc. Natl. Acad. Sci. USA.

[8] Acharyya A., Ge Y.H., Wu H.F., De Grado W.F., Voelz V.A., Gai F. (2019). Exposing the Nucleation Site in alpha-Helix Folding: A Joint Experimental and Simulation Study. J. Phys. Chem. B.

[9] Neumaier S., Reiner A., Buttner M., Fierz B., Kiefhaber T. (2013). Testing the diffusing boundary model for the helix-coil transition in peptides. Proc. Natl. Acad. Sci. USA.

[10] Mohammed O.F., Jas G.S., Lin M.M., Zewail A.H. (2009). Primary Peptide Folding Dynamics Observed with Ultrafast Temperature Jump. Angew. Chem. Int. Edit..

[11] Lin M.M., Mohammed O.F., Jas G.S., Zewail A.H. (2011). Speed limit of protein folding evidenced in secondary structure dynamics. Proc. Natl. Acad. Sci. USA.

[12] Jas G.S., Kuczera K. (2012). Computer simulations of helix folding in homo- and heteropeptides. Mol. Simulat..

[13] Kuczera K., Jas G.S., Elber R. (2009). Kinetics of Helix Unfolding: Molecular Dynamics Simulations with Milestoning. J. Phys. Chem. A.

[14] Kreuzer S.M., Elber R., Moon T.J. (2012). Early Events in Helix Unfolding under External Forces: A Milestoning Analysis. J. Phys. Chem. B.

[15] Jas G.S., Kuczera K. (2004). Equilibrium structure and folding of a helix-forming peptide: Circular dichroism measurements and replica-exchange molecular dynamics simulations. Biophys. J..

[16] Jas G.S., Hegefeld W.A., Middaugh C.R., Johnson C.K., Kuczera K. (2014). Detailed microscopic unfolding pathways of an alpha-helix and a beta-hairpin: Direct observation and molecular dynamics. J. Phys. Chem. B.

[17] Jas G.S., Kuczera K. (2018). Helix-Coil Transition Courses Through Multiple Pathways and Intermediates: Fast Kinetic Measurements and Dimensionality Reduction. J. Phys. Chem. B.

[18] Hegefeld W.A., Chen S.E., DeLeon K.Y., Kuczera K., Jas G.S. (2010). Helix Formation in a Pentapeptide Experiment and Force-field Dependent Dynamics. J. Phys.Chem. A.

[19] Jas G.S., Middaugh C.R., Kuczera K. (2014). Non-Exponential Kinetics and a Complete Folding Pathway of an alpha-Helical Heteropeptide: Direct Observation and Comprehensive Molecular Dynamics. J. Phys. Chem. B.

[20] Jas G.S., Childs E.W., Kuczera K. (2019). Kinetic pathway analysis of an alpha-helix in two protonation states: Direct observation and optimal dimensionality reduction. J. Chem. Phys..

[21] Jas G.S., Kuczera K. (2018). Deprotonation of a Single Amino Acid Residue Induces Significant Stability in an alpha-Helical Heteropeptide. J. Phys. Chem. B.

[22] Chakrabartty A., Kortemme T., Baldwin R.L. (1994). Helix Propensities of the Amino-Acids Measured in Alanine-Based Peptides without Helix-Stabilizing Side-Chain Interactions. Protein Sci..

[23] Scholtz J.M., Qian H., York E.J., Stewart J.M., Baldwin R.L. (1991). Parameters of helix-coil transition theory for alanine-based peptides of varying chain lengths in water. Biopolymers.

[24] Scholtz J.M., Marqusee S., Baldwin R.L., York E.J., Stewart J.M., Santoro M., Bolen D.W. (1991). Calorimetric determination of the enthalpy change for the alpha-helix to coil transition of an alanine peptide in water. Proc. Natl. Acad. Sci. USA.

[25] Chakrabartty A., Baldwin R.L. (1995). Stability of alpha-helices. Adv. Protein Chem..

[26] Baldwin R.L. (1995). Alpha-helix formation by peptides of defined sequence. Biophys. Chem..

[27] Brooks B.R., Brooks C.L., Mackerell A.D., Nilsson L., Petrella R.J., Roux B., Won Y., Archontis G., Bartels C., Boresch S. (2009). CHARMM: The Biomolecular Simulation Program. J. Comput. Chem..

[28] Kutzner C., Pall S., Fechner M., Esztermann A., de Groot B.L., Grubmuller H. (2015). Best bang for your buck: GPU nodes for GROMACS biomolecular simulations. J. Comput. Chem..

[29] Pronk S., Pall S., Schulz R., Larsson P., Bjelkmar P., Apostolov R., Shirts M.R., Smith J.C., Kasson P.M., van der Spoel D. (2013). GROMACS 4.5: A high-throughput and highly parallel open source molecular simulation toolkit. Bioinformatics.

[30] Huang J., Rauscher S., Nawrocki G., Ran T., Feig M., de Groot B.L., Grubmuller H., MacKerell A.D. (2017). CHARMM36m: An improved force field for folded and intrinsically disordered proteins. Nat. Methods.

[31] Jorgensen W.L., Chandrasekhar J., Madura J.D., Impey R.W., Klein M.L. (1983). Comparison of Simple Potential Functions for Simulating Liquid Water. J. Chem. Phys..

[32] Darden T., Perera L., Li L.P., Pedersen L. (1999). New tricks for modelers from the crystallography toolkit: The particle mesh Ewald algorithm and its use in nucleic acid simulations. Struct. Fold. Des..

[33] Kube S., Weber M. (2007). A coarse graining method for the identification of transition rates between molecular conformations. J. Chem. Phys..

[34] Hummer G., Szabo A. (2015). Optimal Dimensionality Reduction of Multistate Kinetic and Markov-State Models. J. Phys. Chem. B.

[35] Senne M., Trendelkamp-Schroer B., Mey A.S.J.S., Schutte C., Noe F. (2012). EMMA: A Software Package for Markov Model Building and Analysis. J. Chem. Theory Comput..

[36] Lopez M.M., Chin D.H., Baldwin R.L., Makhatadze G.I. (2002). The enthalpy of the alanine peptide helix measured by isothermal titration calorimetry using metal-binding to induce helix formation. Proc. Natl. Acad. Sci. USA.

[37] Shi Z.S., Olson C.A., Rose G.D., Baldwin R.L., Kallenbach N.R. (2002). Polyproline II structure in a sequence of seven alanine residues. Proc. Natl. Acad. Sci. USA.

[38] Lednev I.K., Karnoup A.S., Sparrow M.C., Asher S.A. (1999). Nanosecond UV resonance Raman examination of initial steps in alpha-helix secondary structure evolution. J. Am. Chem. Soc..

[39] Matthes D., de Groot B.L. (2009). Secondary Structure Propensities in Peptide Folding Simulations: A Systematic Comparison of Molecular Mechanics Interaction Schemes. Biophys. J..

[40] Freddolino P.L., Park S., Roux B., Schulten K. (2009). Force field bias in protein folding simulations. Biophys. J..

[41] Lindorff-Larsen K., Maragakis P., Piana S., Eastwood M.P., Dror R.O., Shaw D.E. (2012). Systematic validation of protein force fields against experimental data. PLoS ONE.

[42] Robustelli P., Piana S., Shaw D.E. (2018). Developing a molecular dynamics force field for both folded and disordered protein states. Proc. Natl. Acad Sci. USA.

[43] Garcia A.E., Sanbonmatsu K.Y. (2002). Alpha-helical stabilization by side chain shielding of backbone hydrogen bonds. Proc. Natl. Acad. Sci. USA.

[44] Majek P., Elber R. (2010). Milestoning without a Reaction Coordinate. J. Chem. Theory Comput..

[45] Ma P., Cardenas A.E., Chaudhari M.I., Elber R., Rempe S.B. (2017). The Impact of Protonation on Early Translocation of Anthrax Lethal Factor: Kinetics from Molecular Dynamics Simulations and Milestoning Theory. J. Am. Chem. Soc..

[46] Aristoff D., Bello-Rivas J.M., Elber R. (2016). A Mathematical Framework for Exact Milestoning. Multiscale Model. Simul..

[47] Bowman G.R., Noeé F., Pande V.S. (2014). An Introduction to Markov State Models and Their Application to Long Timescale Molecular Simulation. Advances in Experimental Medicine and Biology.

[48] Scherer M.K., Trendelkamp-Schroer B., Paul F., Perez-Hernandez G., Hoffmann M., Plattner N., Wehmeyer C., Prinz J.H., Noe F. (2015). PyEMMA 2: A Software Package for Estimation, Validation, and Analysis of Markov Models. J. Chem. Theory Comput..

[49] Harrigan M.P., Sultan M.M., Hernandez C.X., Husic B.E., Eastman P., Schwantes C.R., Beauchamp K.A., McGibbon R.T., Pande V.S. (2017). MSMBuilder: Statistical Models for Biomolecular Dynamics. Biophys. J..

