# Supplementary material for: Length Dependent Folding Kinetics of Alanine-Based Helical Peptides from Optimal Dimensionality Reduction
Source: Life (Basel). 2021 Apr 24;11(5):385. doi: 10.3390/life11050385 (PMC8170890; doi:10.3390/life11050385)
Supplement: Supplementary file 1 [file life-11-00385-s001.zip › life-11-00385-s001.pdf]

Supplementary Materials

Length Dependent Folding Kinetics of Alanine-Based Helical Peptides from Optimal Dimensionality Reduction

Krzysztof Kuczera[1,2], Robert Szoszkiewicz[3], Jinyan He[1] and Gouri S. Jas[4]

Contents:

MD trajectory data …. 1
Kinetic models ….13
References ….37

MD trajectory lengths:

ALA21 – 2 x 20 μs
ALA15 – 2 x 10 μs
ALA8 – 2 x 10 μs
ALA5 – 2 x 5 μs

System Composition

Table S1. System compositions.
Pep – peptide atoms, Tot – total atoms, Wat – water molecules, Na+ - sodium ions, Cl- - chloride ions, a – cubic box size, nm. h – trajectory started from helix, e – trajectory started from extended conformation.

System	Pep	Tot	Wat	Na+	Cl-	a
ALA5 h	59	2966	967	3	3	3.08
ALA5 e	59	3734	1223	3	3	3.33
ALA8 h	89	4162	1355	4	4	3.44
ALA8 e	89	4585	1496	4	4	3.57
ALA15 h	159	8583	2726	8	8	4.36
ALA15 e	159	8866	2897	8	8	4.45
ALA21 h	219	12077	3942	16	16	4.93
ALA21 e	219	12032	3927	16	16	4.92

Helix content and folding free energy

Table S2.
Average helix content. Fraction of alpha-helical conformations as measured by alpha-helical hydrogen bonds (HB), backbone dihedrals (PP) and DSSP algorithm (see Methods for details)

System	HB	PP	DSSP
ALA 5 h	0.026 ± 0.006	0.090 ± 0.005	-
ALA5 e	0.032 ± 0.006	0.096 ± 0.004	-
ALA5 a+h	0.029 ± 0.004	0.093 ± 0.003	-
ALA8 h	0.060 ± 0.021	0.117 ± 0.019	0.04
ALA8 e	0.068 ± 0.020	0.122 ± 0.016	0.05
ALA8 h+e	0.063 ± 0.014	0.119 ± 0.012	0.05
ALA15 h	0.219 ± 0.132	0.254 ± 0.112	0.21
ALA15 e	0.278 ± 0.116	0.306 ± 0.099	0.27
ALA15 h+e	0.249 ± 0.087	0.280 ± 0.075	0.24
ALA21 h	0.590 ± 0.099	0.584 ± 0.085	0.58
ALA21 e	0.585 ± 0.151	0.578 ± 0.131	0.60
ALA21 h+e	0.588 ± 0.090	0.581 ± 0.078	0.59

Using HB and PP results from the joint trajectories, our populations yield free energies

$$\Delta G = -RT\ ln\frac{f}{1-f}$$

f is the helix fraction, R the gas constant and T=300 K the temperature

ΔG = 2.09$\pm$0.09 and 1.36$\pm$0.03 kcal/mol for ALA5
ΔG = 1.59$\pm$0.14 and 1.19$\pm$0.07 kcal/mol for ALA8
ΔG = 0.66$\pm$0.28 and 0.56$\pm$0.22 kcal/mol for ALA15
ΔG = -0.20$\pm$0.22 and -0.20$\pm$0.19 kcal/mol for ALA21

RMSD clustering analysis

Trajectory structures were clustered by CA atom RMSD using the gromos algorithm to analyze the structures explored and generate trajectory discretization. The results are presented in the table below.

Table S3. Numbers of clusters N_c based on CA atom RMSD as a function of cluster radius R.

	h	e	h+e	h	e	h+e	h	e	h+e	h	e	h+e
ALA5	R=0.20 nm			R=0.15 nm			R=0.10 nm			R=0.08 nm		
N_c	5	5	5	10	9	9	28	28	30	62	62	62

ALA8	R=0.30 nm			R=0.25 nm			R=0.20 nm			R=0.15 nm		
N_c	8	7	8	19	17	19	60	58	63	278	266	305
ALA15	R=0.50 nm			R=0.40 nm			R=0.35 nm			R=0.30 nm		
N_c	10	12	11	42	41	45	124	121	134	450	432	491
ALA21	R=0.55 nm			R=0.50 nm			R=0.45 nm			R=0.40 nm		
N_c	31	33	34	66	71	76	159	174	194	491	526	605

This data shows that the two ALA5 trajectories are essentially sampling the same set of conformations. In contrast, for ALA8, ALA15 and ALA21, the number of clusters in the joint h+e trajectory tends to be larger than in the separate h or alone, indicating that these trajectories only partly overlap. However, at the intermediate level of resolution, R=0.20-0.25 nm for ALA8, R=0.35-0.40 nm for ALA15 and R=0.45-0.50 nm for ALA21, the simulations appear to be converged.

Helical patterns and convergence

Another way of analyzing explored conformations is to consider every helical hydrogen bond as being formed or broken, or, alternatively as every residue as being in or out the alpha-helix region of the Ramachandran map. For a system with n objects in two states we have 2^n total system states. The trajectory results are presented in the table below.

Table S4. Fraction of possible patterns sampled in the trajectories. For each peptide, the fractions defined as $f = N_{patt}/2^n$ are calculated, with the observed number of patterns and n the number of objects. Hydrogen bonds (HB) are considered formed if the distance between the peptide oxygen atom of residue i is within 3.6 Å of the peptide N atom of residue i+4 and broken otherwise. A residue is considered to be in the helical region of the Ramachandran map if its backbone dihedral angles (PP) are within 20° of the ideal helix conformation, (ϕ,ψ) =(-62°,-41°).

	h	e	h+e	h	e	h+e
ALA5	HB n=3			PP n=5		
	1.000	1.000	1.000	1.000	1.000	1.000
ALA8	HB n=6			PP n=8		
	1.000	1.000	1.000	1.000	1.000	1.000
ALA15	HB n=13			PP n=15		
	0.3928	0.4567	0.5146	0.6065	0.6451	0.7035
ALA21	HB n=19			PP n=21		

	0.0665	0.0575	0.0868	0.1247	0.1144	0.1671

These results are qualitatively similar to those of the RMSD clustering. It appears that all possible patterns are sampled in each of the h and e trajectories for the shorter peptides, ALA5 and ALA8. For the longer systems, ALA15 and ALA21, only partial pattern sampling is detected, indicating a non-random distribution of conformers. Additionally, the partial overlap of h and e trajectory structures is confirmed for ALA15 and ALA21.

Relaxation times from MD simulations

Autocorrelation functions for various quantities x(t) were calculated as

$$C(t) = \langle \Delta x(t) \Delta x(0) \rangle / \langle \Delta x(0)^2 \rangle \text{ with } \Delta x(t) = x(t) - \langle x \rangle$$

Time scales were extracted from ACFs by fitting to a two-exponential decay

$$C(t) = a_0 e^{-t/\tau_1} + (1 - a_0) e^{-t/\tau_2}$$

The slower relaxation times τ_2 were used to calibrate ODR kinetic simulations.

Table S5. Results of two-exponential fits from peptide simulations. EEDIST – end-to end distance, RGYR – radius of gyration, RMSDH – CA RMSD from ideal helix, SASA – solvent accessible surface area, VOLU – volume, HBACNT – number of formed helical hydrogen bonds, PPACNT – number of residues in helical region of Ramachandran map.

ALA5	h			e		
	a_0	τ_1, ns	τ_2, ns	a_0	τ_1, ns	τ_2,ns
EEDIST	0.50	0.24	2.0	0.44	0.44	1.9
RGYR	0.47	0.30	2.3	0.34	0.20	2.0
RMSDH	0.41	0.38	2.7	0.22	0.14	2.0
SASA	0.47	0.02	2.1	0.41	0.01	1.9
VOLU	0.70	0.01	2.2	0.64	0.01	2.0
HBACNT	0.42	0.06	3.2	0.27	0.02	2.1
PPACNT	0.62	0.01	2.8	0.63	0.01	2.0

Table S6. Results of two-exponential fits from peptide simulations. EEDIST – end-to end distance, RGYR – radius of gyration, RMSDH – CA RMSD from ideal helix, SASA – solvent accessible surface area, VOLU – volume, HBACNT – number of formed helical hydrogen bonds, PPACNT – number of residues in helical region of Ramachandran map.

ALA8	h			e		
	a_0	τ_1, ns	τ_2, ns	a_0	τ_1, ns	τ_2,ns
EEDIST	0.78	0.46	11.7	0.69	0.41	10.0
RGYR	0.69	1.2	16.0	0.50	0.64	11.7
RMSDH	0.20	0.92	12.7	0.11	0.19	14.6
SASA	0.51	0.98	14.0	0.34	0.37	12.7

VOLU	0.50	0.06	11.7	0.44	0.02	12.9
HBACNT	0.11	0.08	12.0	0.06	0.01	14.8
PPACNT	0.30	0.04	12.1	0.24	0.01	14.3

Table S7. Results of two-exponential fits from peptide simulations. EEDIST – end-to end distance, RGYR – radius of gyration, RMSDH – CA RMSD from ideal helix, SASA – solvent accessible surface area, VOLU – volume, HBACNT – number of formed helical hydrogen bonds, PPACNT – number of residues in helical region of Ramachandran map.

ALA15	h			e		
	a_0	τ_1, ns	τ_2, ns	a_0	τ_1, ns	τ_2,ns
EEDIST	0.78	0.34	7.7	0.74	0.38	7.0
RGYR	0.52	2.0	125	0.50	2.1	77
RMSDH	0.08	0.50	123	0.06	0.35	89
SASA	0.22	1.5	128	0.19	1.4	83
HBACNT	0.00	-	130	0.00	-	88
PPACNT	0.00	-	125	0.00	-	85

Table S8. Results of two-exponential fits from peptide simulations. EEDIST – end-to end distance, RGYR – radius of gyration, RMSDH – CA RMSD from ideal helix, SASA – solvent accessible surface area, VOLU – volume, HBACNT – number of formed helical hydrogen bonds, PPACNT – number of residues in helical region of Ramachandran map.

ALA21	h			e		
	a_0	τ_1, ns	τ_2, ns	a_0	τ_1, ns	τ_2,ns
EEDIST	0.74	1.2	172	0.86	2.0	362
RGYR	0.56	3.2	134	0.81	8.1	602
RMSDH	0.07	0.67	233	0.32	43.4	760
SASA	0.16	1.1	179	0.42	13.2	793
HBACNT	0.02	0.02	218	0.11	16.7	619
PPACNT	0.02	0.01	221	0.14	13.1	631

Table S9. Slowest relaxation times τ_2 in MD trajectories. Units: ns.
This is a summary of data from tables S5-S8.
Trajectory *h* started from helix, trajectory *e* from extended conformation

System	Trajectory h	Trajectory e	Average
ALA21	200-230	600-800	400-500
ALA15	120-130	80-90	100-110
ALA8	12-16	10-15	11-16
ALA5	2.0-3.2	1.9-2.1	2.0-2.6

Autocorrelation functions from MD trajectories

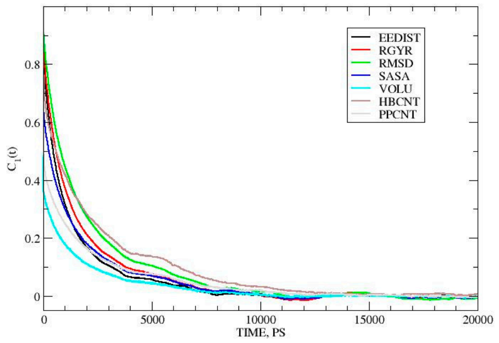

A. ACFs from ALA5 traj. h

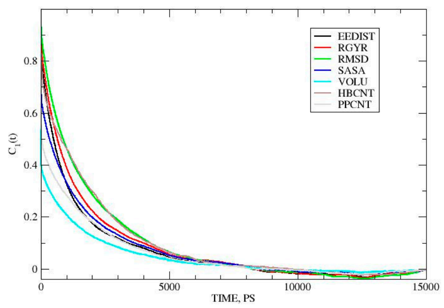

B. ACFs from ALA5 traj. e

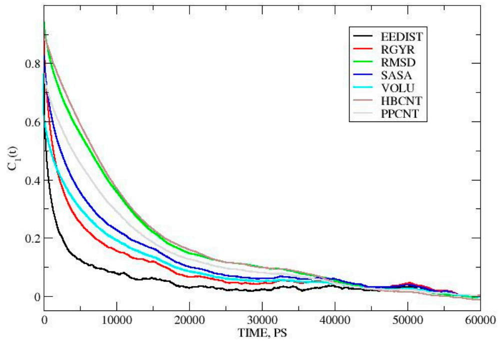

A. ACFs from ALA8 traj. h

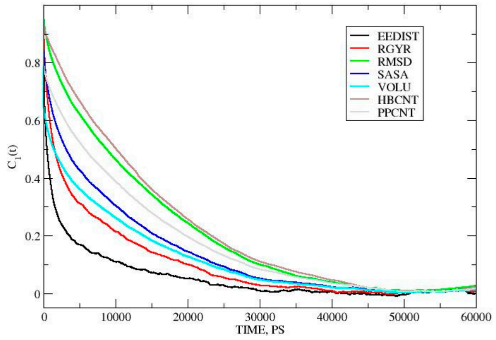

B. ACFs from ALA8 traj. e

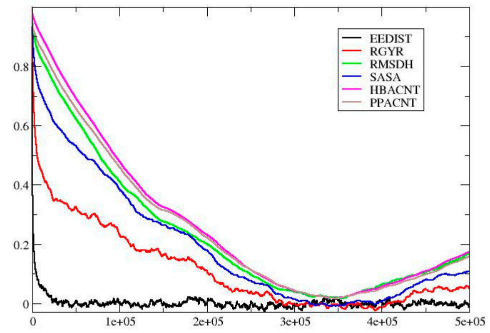

A. ACFs from ALA15 traj. h

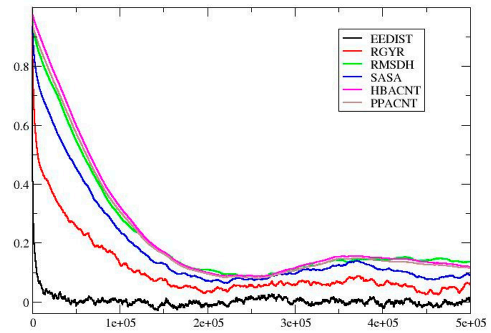

B. ACFs from ALA15 traj. e

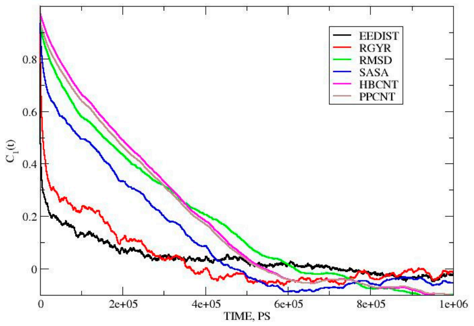

A. ACFs from ALA21 traj. h

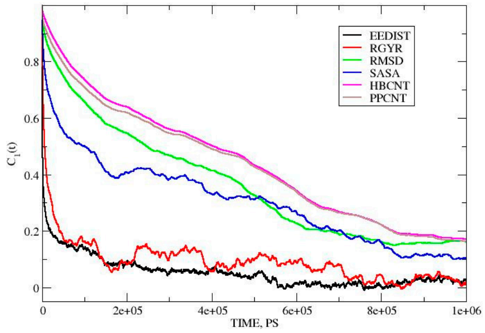

B. ACFs from ALA21 traj. e

Helix folding and unfolding in MD

Rate constants for folding (k_f) and unfolding (k_u) were calculated using a two-state model, using the equilibrium constant K and slow relaxation time τ_2.

$$K = \frac{k_f}{k_u} \ \ and \ \ k_f + k_u = \frac{1}{\tau_2}$$

So that

$$k_f = \frac{1}{\tau_2}\frac{1}{1+K} \ \ and \ k_u = \frac{1}{\tau_2}\frac{K}{1+K}$$

Using K=f(1-f) and τ_2 data based on HB and PP populations and relaxation rates in the h and e trajectories yielded four estimates of the rates for each peptide, allowing the estimates of average values and error presented below.

Table S10. Average folding and unfolding rates with 95% confidence intervals. Units: ns^{-1}.

System	k_u	k_f
ALA5	$(3.86 \pm 1.13) \times 10^{-1}$	$(2.55 \pm 0.24) \times 10^{-2}$
ALA8	$(6.90 \pm 0.11) \times 10^{-2}$	$(6.90 \pm 0.36) \times 10^{-3}$
ALA15	$(7.13 \pm 0.22) \times 10^{-3}$	$(2.57 \pm 0.84) \times 10^{-3}$
ALA21	$(1.27 \pm 0.97) \times 10^{-3}$	$(1.80 \pm 1.38) \times 10^{-3}$

Structural change in course of MD: RMSD and hydrogen bond count

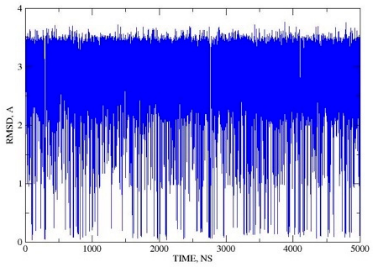

A. CA RMSD from helix: ALA5 h

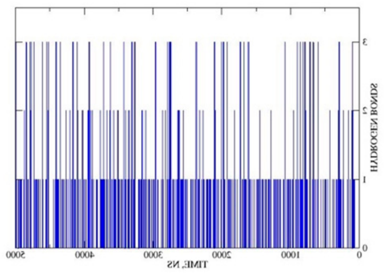

B. Hydrogen bond count: ALA5 h

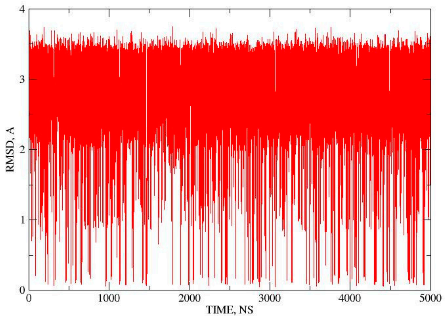

C. CA RMSD from helix: ALA5 e

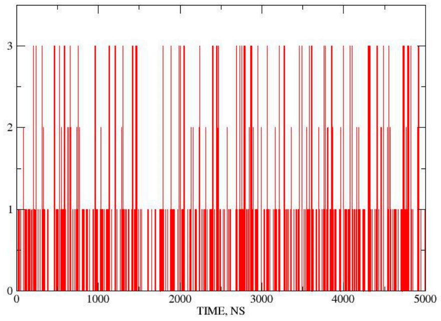

D. Hydrogen bond count: ALA5 e

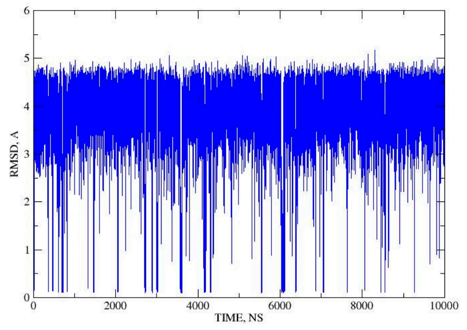

A. CA RMSD from helix: ALA8 h

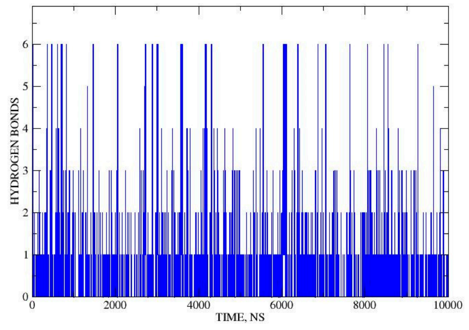

B. Hydrogen bond count: ALA8 h

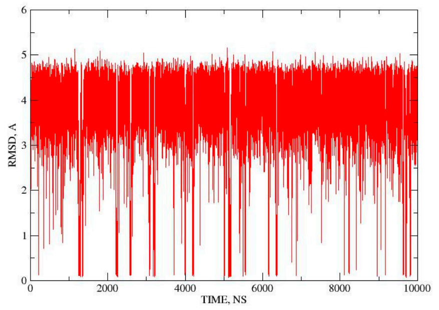

C. CA RMSD from helix: ALA8 e

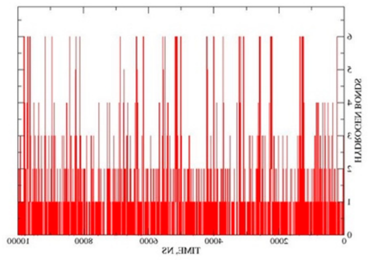

D. Hydrogen bond count: ALA8 e

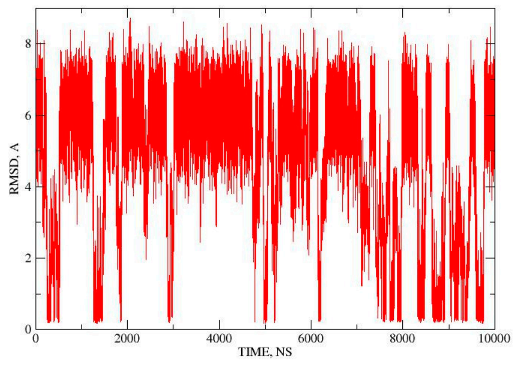

C. CA RMSD from helix: ALA15 e

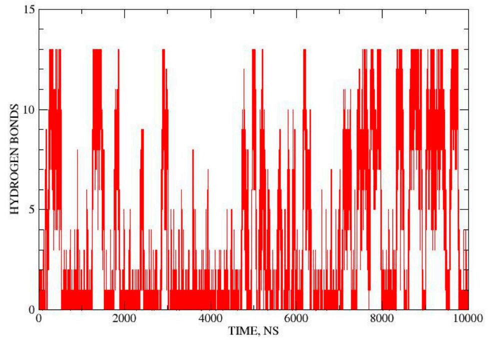

D. Hydrogen bond count: ALA15 e

Results for ALA15 trajectory h are presented in main text.

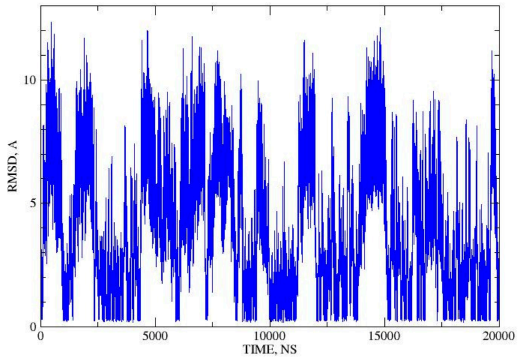	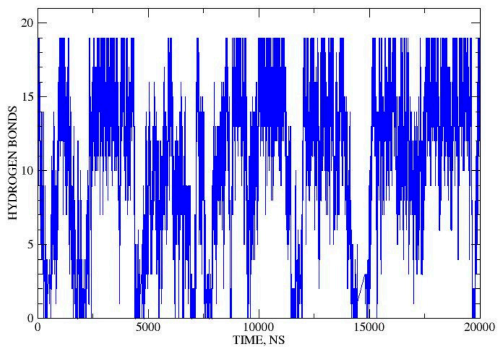
A. CA RMSD from helix: ALA21 h	B. Hydrogen bond count: ALA21 h
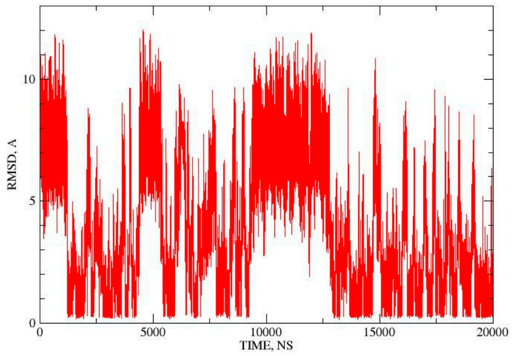	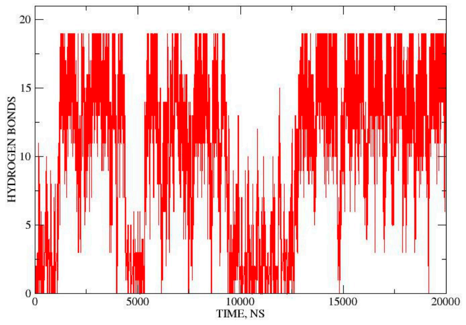
C. CA RMSD from helix: ALA21 e	D. Hydrogen bond count: ALA21 e

Helix folding statistics

Hydrogen bond populations P(HBi,NHB). In these tables the columns correspond to populations of individual hydrogen bonds HBi, i=1,2,…,n-2 (n = number of residues). The rows correspond to different data subsets extracted from the MD sumulations, NHB=0,1,…,n-2, where NHB is the total number of hydrogen bonds in the peptide structure. Thus, the NHB=0 row corresponds to a sample of structures with 0 total hydrogen bonds, so all populations are 0 in all systems, while the NHB=3 row for ALA5 corresponds to a subset of structures with a total 3 hydrogen bonds formed (i.e. all possible for this peptide), so all populations are 1 there. Similarly the NHB=1 row for ALA5 corresponds to systems with 1 formed hydrogen bond, so if the h-bonds were randomly distributed, all populations should be 1/3 = 0.33, but they are 0.35, 0.18 and 0.46 for HB1, HB2 and HB3, respectively, indicating marked preference for initiation at HB1 (N-terminus) and even higher at HB3 (C-terminus).

ALA5

0.00 0.00 0.00 0.00
1.00 0.35 0.18 0.46
2.00 0.49 0.90 0.60
3.00 1.00 1.00 1.00

ALA8

0.00 0.00 0.00 0.00 0.00 0.00 0.00
1.00 0.24 0.12 0.15 0.11 0.12 0.24
2.00 0.32 0.38 0.27 0.26 0.43 0.35
3.00 0.39 0.48 0.61 0.62 0.49 0.39
4.00 0.49 0.63 0.95 0.93 0.58 0.41
5.00 0.65 0.95 0.96 0.96 0.94 0.54
6.00 1.00 1.00 1.00 1.00 1.00 1.00

ALA15

0.00 0.00 0.00 0.00 0.00 0.00 0.00 0.00 0.00 0.00 0.00 0.00 0.00 0.00
1.00 0.13 0.07 0.08 0.07 0.06 0.08 0.07 0.08 0.06 0.07 0.06 0.07 0.12
2.00 0.15 0.17 0.18 0.15 0.13 0.15 0.13 0.13 0.13 0.15 0.14 0.20 0.17
3.00 0.20 0.26 0.29 0.21 0.22 0.24 0.26 0.24 0.22 0.22 0.28 0.22 0.17
4.00 0.19 0.23 0.31 0.39 0.35 0.39 0.39 0.38 0.33 0.36 0.30 0.21 0.17
5.00 0.19 0.23 0.30 0.41 0.55 0.51 0.55 0.56 0.55 0.42 0.34 0.23 0.16
6.00 0.18 0.24 0.33 0.41 0.55 0.67 0.69 0.73 0.66 0.59 0.45 0.31 0.21
7.00 0.25 0.32 0.41 0.52 0.66 0.77 0.93 0.77 0.68 0.59 0.48 0.35 0.26
8.00 0.27 0.33 0.49 0.59 0.75 0.95 0.96 0.95 0.78 0.68 0.53 0.42 0.30
9.00 0.31 0.40 0.57 0.69 0.96 0.97 0.98 0.97 0.95 0.75 0.62 0.46 0.36
10.00 0.41 0.51 0.74 0.96 0.97 0.98 0.98 0.98 0.97 0.94 0.68 0.52 0.35
11.00 0.47 0.60 0.97 0.98 0.98 0.98 0.98 0.98 0.98 0.97 0.94 0.65 0.49
12.00 0.71 0.96 0.97 0.98 0.98 0.98 0.98 0.98 0.98 0.98 0.96 0.94 0.57
13.00 1.00 1.00 1.00 1.00 1.00 1.00 1.00 1.00 1.00 1.00 1.00 1.00 1.00

ALA21

0.00 0.00 0.00 0.00 0.00 0.00 0.00 0.00 0.00 0.00 0.00 0.00 0.00 0.00 0.00 0.00 0.00 0.00 0.00 0.00
1.00 0.09 0.04 0.06 0.04 0.05 0.07 0.04 0.04 0.06 0.05 0.04 0.05 0.06 0.04 0.06 0.07 0.04 0.04 0.08
2.00 0.11 0.11 0.11 0.11 0.11 0.11 0.10 0.11 0.14 0.15 0.11 0.10 0.11 0.10 0.09 0.11 0.07 0.10 0.09
3.00 0.11 0.12 0.16 0.15 0.18 0.17 0.17 0.20 0.21 0.24 0.21 0.16 0.16 0.16 0.18 0.15 0.14 0.09 0.06
4.00 0.10 0.12 0.17 0.23 0.26 0.26 0.28 0.33 0.28 0.33 0.30 0.23 0.23 0.18 0.18 0.18 0.15 0.12 0.08
5.00 0.11 0.15 0.21 0.32 0.35 0.33 0.36 0.37 0.33 0.36 0.35 0.31 0.27 0.25 0.28 0.22 0.18 0.15 0.13
6.00 0.14 0.18 0.24 0.31 0.36 0.39 0.41 0.42 0.41 0.47 0.46 0.43 0.35 0.38 0.33 0.23 0.18 0.15 0.12
7.00 0.15 0.21 0.26 0.32 0.41 0.48 0.54 0.52 0.49 0.54 0.53 0.46 0.46 0.42 0.39 0.30 0.22 0.17 0.14
8.00 0.24 0.28 0.36 0.41 0.48 0.53 0.62 0.67 0.55 0.59 0.56 0.57 0.49 0.45 0.36 0.30 0.23 0.17 0.13
9.00 0.20 0.26 0.35 0.42 0.47 0.52 0.60 0.68 0.72 0.72 0.72 0.65 0.60 0.54 0.48 0.40 0.30 0.21 0.16
10.00 0.24 0.33 0.42 0.49 0.57 0.62 0.70 0.76 0.80 0.88 0.77 0.67 0.61 0.55 0.47 0.40 0.31 0.23 0.17
11.00 0.28 0.35 0.45 0.53 0.60 0.68 0.75 0.81 0.88 0.90 0.90 0.76 0.69 0.60 0.54 0.46 0.36 0.27 0.20
12.00 0.29 0.37 0.49 0.56 0.64 0.72 0.81 0.91 0.90 0.91 0.92 0.89 0.79 0.68 0.60 0.53 0.43 0.34 0.24
13.00 0.32 0.41 0.53 0.61 0.70 0.80 0.94 0.94 0.93 0.93 0.93 0.92 0.91 0.74 0.67 0.56 0.47 0.39 0.29

14.00 0.33 0.42 0.56 0.68 0.79 0.95 0.95 0.95 0.95 0.96 0.96 0.95 0.95 0.93 0.77 0.64 0.52 0.41 0.30
15.00 0.35 0.45 0.64 0.79 0.96 0.96 0.97 0.97 0.97 0.97 0.97 0.97 0.97 0.96 0.95 0.75 0.60 0.45 0.33
16.00 0.40 0.52 0.73 0.97 0.97 0.98 0.98 0.98 0.98 0.98 0.98 0.97 0.97 0.97 0.97 0.95 0.71 0.56 0.41
17.00 0.47 0.63 0.97 0.98 0.98 0.98 0.98 0.98 0.98 0.98 0.98 0.98 0.98 0.98 0.98 0.97 0.94 0.69 0.53
18.00 0.68 0.96 0.98 0.98 0.98 0.99 0.99 0.99 0.99 0.99 0.98 0.99 0.99 0.99 0.98 0.98 0.97 0.95 0.65
19.00 1.00 1.00 1.00 1.00 1.00 1.00 1.00 1.00 1.00 1.00 1.00 1.00 1.00 1.00 1.00 1.00 1.00 1.00 1.00

Hydrogen bond relaxation times: local dynamics

Autocorrelation functions were calculated for all helical hydrogen bonds, and the average function fitted to a two-exponential decay, analogously to the procedure for the global variables described above.

ALA5	h			e		
	a_0	τ_1, ns	τ_2, ns	a_0	τ_1, ns	τ_2,ns
HB average	0.47	0.20	2.2	0.34	0.10	1.8

ALA8	h			e		
	a_0	τ_1, ns	τ_2, ns	a_0	τ_1, ns	τ_2,ns
HB average	0.56	0.95	12	0.45	0.70	13

ALA15	h			e		
	a_0	τ_1, ns	τ_2, ns	a_0	τ_1, ns	τ_2,ns
HB average	0.28	1.8	100	0.24	1.8	77

ALA21	h			e		
	a_0	τ_1, ns	τ_2, ns	a_0	τ_1, ns	τ_2,ns
HB average	0.25	6.8	170	0.25	7.0	200

Data for central hydrogen bonds of the peptides were very similar to the average values presented above.

Kinetic coarse graining with ODR

Detailed coarse graining protocol.[1]

1. Trajectories are clustered by CA atom RMSD using gromos algorithm in GROMACS. Here both h and e trajectories were joined for clustering for each peptide. The number of clusters generated is denoted as N_c.
2. Trajectories are discretized using the Lifetime-Based approach of Hummer and coworkers[2]. Each cluster center and a region within a core radius R_c around it was considered a microstate. A transition between microstate i and j is counted when the trajectory entered the core region of j first after leaving the core region of i. This yielded the raw transition matrix T_{ij}*. The number of microstates n is thus the number of clusters $n=N_c$.
3. The residence time t_i in state i is counted as the time spent within the core of i plus half the time spent in transitions between i and the other states $j \neq i$. The transition matrix was symmetrized $T_{ij} = (T_{ij}^* + T_{ji}^*)$ to obtain the best estimate. [2]The kinetic rate matrix is calculated as $K_{ij} = T_{ij}/t_j$.
4. Microstates are divided into a small number N of aggregate states, sometimes called metastable states in the literature, using the PCCA+ algorithm[3] implemented in the package EMMA 1.4[4]. This algorithm groups states according to the sign structure of the eigenvectors of the Transition matrix T_{ij}, which are the same as the eigenvectors of the kinetic rate matrix K_{ij}.
5. Using a small number of aggregate states, N=2-5, we use the Optimal Dimensionality Reduction method to generate an effective rate matrix **R** which best represents the slowest dynamical processes in the system.[5] The NxN matrix **R** is based on the full N_cxN_c rate matrix **K** and the aggregate state definitions as follows:

$$\boldsymbol{R} = \boldsymbol{P}_{eq}\boldsymbol{1}_N^T - \boldsymbol{D}_N\left[\boldsymbol{A}^T\left(\boldsymbol{p}_{eq}\boldsymbol{1}_n^T - \boldsymbol{K}\right)^{-1}\boldsymbol{D}_n\boldsymbol{A}\right]^{-1}$$

Here $\mathbf{1}_n$ and $\mathbf{1}_N$ are the unit matrices of the higher and lower dimensional space, respectively, $\mathbf{p}_{eq}$ and $\mathbf{P}_{eq}$ are the corresponding diagonal matrices with equilibrium populations, $\mathbf{D}_n$ and $\mathbf{D}_N$ the diagonal matrices of eigenvalues. **K** is the n x n kinetic matrix and **A** is the n x N transformation matrix from higher to lower dimensional space, such that $\mathbf{P}_{eq} = \mathbf{A}^T\mathbf{p}_{eq}$, with T denoting a matrix transpose.[5]

Definitions of quantities presented below:

Aggregate rate matrices **R** and aggregate state properties calculated for N=2-5 aggregate states. Values marked in red are considered too low to be reliable, given the trajectory lengths, and are ignored in analysis.

Off-diagonal elements R_{ij} are rates for j to i transitions, while the diagonal element $-1/R_{ii}$ is the lifetime of state i. R_{ij} values are in ns^{-1}.

Aggregate state properties are:
NHB – number of alpha-helical h-bonds (between C=O of residue i and N-H of residue i+4, including blocking groups, averaged over constituent MD frames;
RMSD – CA atom RMSD from ideal helix, averaged over constituent MD frames;
population – fraction of trajectory time spent in state i;
$\Delta \boldsymbol{G}$ – free energy relative to most populated state (kcal/mol), at T=300 K

$$\Delta G_i = -RT \ln\left(\frac{pop_i}{pop_{max}}\right);$$

Ns – number of original clusters contributing to this aggregate state;
Struc – rough structure characterization of representative structure (the highest populated cluster that is part of this aggregate state) from molecular graphics.
Aggregate RMSD – matrix of aggregate state distances in A.
Diagonal values DA(i,i) give average of CA atom RMSD in A over all pairs of cluster centers belonging to aggregate set i (values of zero correspond to aggregates made up of a single cluster). This is a measure of aggregate state inhomogeneity.
Off-diagonal values DA(i,j) give average of CA atom RMSD in A over all pairs of cluster centers where the first member of the pair belongs to aggregate set i and the second to aggregate set j. This is a measure of average structural distance between aggregate states.

Ala_5 ORD models

Ala5. Number of clusters N_c =5, core radius R_c = 0.06 nm

Table S11a.
Comparison of results from full K matrix (dimension N_c = 5) and reduced dimensionality models with dimensions N=2-5. Relaxation times, ns.

Full K matrix	N=2	N=3	N=4	N=5
2.41	2.37	2.40	2.40	2.41
0.78		0.77	0.77	0.77
0.07			0.07	0.07
0.02				0.02

N=2
... Aggregate rates R

1	-0.028986	0.392077
2	0.028986	-0.392077

#	NHB	RMSD	Population	ΔG	Ns	Struct
1	0.0	2.9	0.9311881	0.0000	3	extended
2	1.0	1.1	0.0688119	1.5530	2	turn/nucleus

N=3
... Aggregate rates R

1	-1.222012	0.114911	0.058338
2	0.162748	-0.402427	0.022427
3	1.059264	0.287517	-0.080765

#	NHB	RMSD	Population	ΔG	Ns	Struct
1	0.0	2.4	0.0485799	1.7286	1	extended
2	1.0	1.1	0.0688119	1.5210	2	turn/nucleus
3	0.0	2.9	0.8826082	0.0000	2	extended

N=4
... Aggregate rates R N=4

1	-1.222478	0.085941	0.114775	0.044488
2	0.522746	-9.977178	0.145320	4.965689
3	0.162556	0.033837	-0.402484	0.016701
4	0.537176	9.857400	0.142388	-5.026878

#	NHB	RMSD	Population	ΔG	Ns	Struct
1	0.0	2.4	0.0485799	1.4855	1	extended
2	0.0	2.8	0.2955044	0.4093	1	extended
3	1.0	1.1	0.0688119	1.2780	2	turn/nucleus
4	0.0	3.0	0.5871038	0.0000	1	extended

N=5

... Aggregate rates R N=5

1	-5.026879	0.152592	0.537159	0.094810	9.857401
2	0.014744	-8.684531	0.148182	38.616596	0.027237
3	0.044486	0.127013	-1.222499	0.057711	0.085942
4	0.001960	8.262917	0.014407	-38.929882	0.006598
5	4.965689	0.142008	0.522752	0.160765	-9.977178

#	NHB	RMSD	Population	ΔG	Ns	Struct
1	0.0	3.0	0.5871038	0.0000	1	extended
2	0.9	1.1	0.0566939	1.3935	1	turn
3	0.0	2.4	0.0485799	1.4855	1	extended
4	1.4	0.9	0.0121180	2.3133	1	helix
5	0.0	2.8	0.2955044	0.4093	1	extended

Ala5. Number of clusters N_c = 9, core radius R_c = 0.05 nm

Table S11b.

Comparison of results from full K matrix (dimension N_c = 9) and reduced dimensionality models with dimensions N=2-5. Relaxation times, ns.

Full K matrix	N=2	N=3	N=4	N=5
2.28	2.26	2.26	2.27	2.26
1.30		1.29	1.29	1.30
0.91			0.90	0.90
0.87				0.87
0.67				

N=2

... Aggregate rates RHS

1	-0.026995	0.416878
2	0.026995	-0.416878

#	NHB	RMSD	Population	ΔG	Ns	Struct
1	0.0	2.9	0.9391831	0.0000	7	extended/PPII
2	1.1	1.0	0.0608169	1.6317	2	helical turn

...Aggregate distance matrix DA(I,J)

2.0	2.6
2.6	1.0

N=3

... Aggregate rates RHS

1	-0.417578	0.108605	0.026611
2	0.008784	-0.769644	0.003480
3	0.408795	0.661039	-0.030091

#	NHB	RMSD	Population	ΔG	Ns	Struct
1	1.1	1.0	0.0608169	1.6286	2	helical turn
2	0.3	2.2	0.0049120	3.1285	1	intermediate*
3	0.0	2.9	0.9342711	0.0000	6	extended/PPII

...Aggregate distance matrix DA(I,J) in A

1.0	2.3	2.6
0.0	0.0	2.4
0.0	0.0	1.9

N=4

... Aggregate rates RHS

1	-0.144142	0.908115	0.571991	0.281888
2	0.119958	-0.985182	0.089647	0.130888
3	0.003409	0.004045	-0.769652	0.008736
4	0.020774	0.073021	0.108014	-0.421511

#	NHB	RMSD	Population	ΔG	Ns	Struct
1	0.0	2.9	0.8252453	0.0000	5	extended/PPII
2	0.1	2.4	0.1090258	1.2066	1	C-terminal turn
3	0.3	2.2	0.0049120	3.0546	1	intermediate*
4	1.1	1.0	0.0608169	1.5546	2	helical turn

...Aggregate distance matrix DA(I,J)

1.7	2.2	2.4	2.7
2.2	0.0	2.0	2.2
2.4	2.0	0.0	2.3
2.7	2.2	2.3	1.0

N=5

... Aggregate rates RHS

1	-1.103311	0.152931	0.026576	0.039681	0.031783
2	0.026441	-0.772445	0.004144	0.008107	0.002638
3	0.101838	0.091845	-0.985260	0.131382	0.120565
4	0.084831	0.100244	0.073297	-0.423258	0.018630
5	0.890201	0.427425	0.881242	0.244088	-0.173617

#	NHB	RMSD	Population	ΔG	Ns	Struct
1	0.0	2.2	0.0284419	1.9867	1	turn
2	0.3	2.2	0.0049120	3.0337	1	intermediate*
3	0.1	2.4	0.1090258	1.1857	1	C-terminal turn
4	1.1	1.0	0.0608169	1.5337	2	helical turn
5	0.0	3.0	0.7968034	0.0000	4	extended/PPII

*intermediate: acetyl C=O interacting with backbone N-H groups of Ala 3, 4, and 5.

...Aggregate distance matrix DA(I,J)

0.0	1.5	1.9	1.8	2.2
1.5	0.0	2.0	2.3	2.7
1.9	2.0	0.0	2.2	2.3
1.8	2.3	2.2	1.0	3.0
2.2	2.7	2.3	3.0	1.4

Ala5. Number of clusters N_c = 30, core radius R_c = 0.025 nm

Table S11c.
Comparison of results from full K matrix (dimension N_c = 30) and reduced dimensionality models with dimensions N=2-5. Relaxation times, ns.

Full K matrix	N=2	N=3	N=4	N=5
2.12	2.03	2.03	2.03	2.03
1.63		1.62	1.63	1.63
1.01			1.00	1.00
1.00				0.89
1.00				

N=2

... Aggregate rates RHS

1	-0.466025	0.026655
2	0.466025	-0.026655

#	NHB	RMSD	Population	ΔG	Ns	Struct
1	1.2	0.8	0.0541009	1.7057	6	helix
2	0.0	2.9	0.9458991	0.0000	24	extended/PPII

...Aggregate distance matrix DA(I,J)

1.9	2.2
2.2	1.9

N=3

... Aggregate rates RHS

1	-0.611422	0.001206	0.004937
2	0.499442	-0.026660	0.464635
3	0.111980	0.025454	-0.469572

#	NHB	RMSD	Population	ΔG	Ns	Struct
1	0.6	2.1	0.0022830	3.5927	1	nucleus
2	0.0	2.9	0.9458991	0.0000	24	extended/PPII
3	1.2	0.8	0.0518179	1.7314	5	alpha

...Aggregate distance matrix DA(I,J)

0.0	2.2	2.4
2.2	1.9	2.2
2.4	2.2	1.6

N=4

... Aggregate rates RHS

1	-0.611422	0.000647	0.004937	0.001207
2	0.000284	-0.995882	0.000080	0.001051
3	0.111975	0.004152	-0.469580	0.025477
4	0.499163	0.991083	0.464563	-0.027734

#	NHB	RMSD	Population	ΔG	Ns	Struct
1	0.6	2.1	0.0022830	3.5920	1	nucleus
2	0.0	2.0	0.0010000	4.0841	1	turn(1-2)
3	1.2	0.8	0.0518179	1.7308	5	alpha
4	0.0	2.9	0.9448991	0.0000	23	extended/PPII

...Aggregate distance matrix DA(I,J)

0.0	2.3	2.4	2.2
2.3	0.0	1.6	1.8
2.4	1.6	1.6	2.2
2.2	1.8	2.2	1.9

N=5

... Aggregate rates RHS

1	-1.124412	-0.000916	-0.000261	0.000697	0.001814
2	-0.000591	-0.995886	0.000282	0.000080	0.001053
3	-0.000384	0.000644	-0.611424	0.004937	0.001210
4	0.023278	0.004147	0.111973	-0.469581	0.025480
5	1.102109	0.992011	0.499430	0.463866	-0.029557

#	NHB	RMSD	Population	ΔG	Ns	Struct
1	0.3	2.1	0.0015500	3.8219	1	turn(2-3)
2	0.0	2.0	0.0010000	4.0831	1	turn(1-2)
3	0.6	2.1	0.0022830	3.5910	1	nucleus*
4	1.2	0.8	0.0518179	1.7298	5	alpha
5	0.0	2.9	0.9433491	0.0000	22	extended/PPII

...Aggregate distance matrix DA(I,J)
0.0 1.7 1.7 2.1 1.8
1.7 0.0 2.3 1.6 1.8
1.7 2.3 0.0 2.4 2.2
2.1 1.6 2.4 1.6 2.2
1.8 1.8 2.2 2.2 1.9

*nucleus state with bifurcated h-bonds from ACEO to HN3 and HN5

Ala5. Number of clusters N_c = 62, core radius R_c = 0.02 nm

Table S11d.
Comparison of results from full K matrix (dimension N_c = 62) and reduced dimensionality models with dimensions N=2-5. Relaxation times, ns.

Full K matrix	N=2	N=3	N=4	N=5
2.26	2.15	2.15	2.15	2.15
1.67		1.64	1.64	1.64
1.29			1.23	1.25
1.11				1.08
1.07				

N=2
... Aggregate rates RHS
1 -0.441252 0.024558
2 0.441252 -0.024558

#	NHB	RMSD	Population	ΔG	Ns	Struct
1	1.2	0.8	0.0527299	1.7219	7	helix/1…5 nucleus*
2	0.0	2.9	0.9472701	0.0000	55.	Extended/PPII

...Aggregate distance matrix DA(I,J)
1.6 2.3
2.3 1.8

N=3
... Aggregate rates RHS
1 -0.605761 0.000947 0.003587
2 0.505017 -0.024057 0.438920
3 0.100745 0.023110 -0.442506

NHB RMSD Population ΔG Ns Struct

1	0.7	2.1	0.0017780	3.7432	1	1…5 nucleus*
2	0.0	2.9	0.9482851	0.0000	56	extended/PPII
3	1.3	0.8	0.0499369	1.7549	5	helix

...Aggregate distance matrix DA(I,J)

0.0	2.2	2.4
2.2	1.9	2.2
2.4	2.2	1.2

N=4

1	-0.605763	-0.000514	0.003585	0.000949
2	-0.000350	-0.811425	-0.000192	0.001047
3	0.100707	-0.007912	-0.442535	0.023151
4	0.505407	0.819851	0.439142	-0.025147

#	NHB	RMSD	Population	ΔG	Ns	Struct
1	0.7	2.1	0.0017780	3.7424	1	1…5 nucleus*
2	0.3	2.1	0.0012080	3.9728	1	extended/PPII
3	1.3	0.8	0.0499369	1.7542	5	helix
4	0.0	2.9	0.9470771	0.0000	55	extended

...Aggregate distance matrix DA(I,J)

0.0	1.9	2.4	2.2
1.9	0.0	2.1	1.9
2.4	2.1	1.2	2.2
2.2	1.9	2.2	1.9

N=5

... Aggregate rates RHS

1	-0.605765	-0.000445	-0.000573	0.003584	0.000951
2	-0.000303	-0.812945	0.034198	-0.000158	0.000994
3	-0.000491	0.043058	-0.910072	-0.000215	0.001424
4	0.100663	-0.006527	-0.007048	-0.442566	0.023200
5	0.505896	0.776859	0.883495	0.439355	-0.026569

#	NHB	RMSD	Population	ΔG	Ns	Struct
1	0.7	2.1	0.0017780	3.7415	1	1…5 nucleus
2	0.3	2.1	0.0012080	3.9719	1	turn/extended
3	0.1	2.5	0.0015240	3.8334	2	turn
4	1.3	0.8	0.0499369	1.7532	5	helix
5	0.0	2.9	0.9455531	0.0000	53	extended

*1…5 nucleus – ALA1 C=O…ALA5 N-H hydrogen bond

...Aggregate distance matrix DA(I,J)

0.0	1.9	1.2	2.4	2.3
1.9	0.0	2.1	2.1	1.9
1.2	2.1	1.8	2.2	2.1

2.4	2.1	2.2	1.2	2.2
2.3	1.9	2.1	2.2	1.8

Ala_8 ODR results

2 x 5 μs MD trajectories at 300 K
Discretization by clustering with CA atom RMSD for number of clusters N_c = 8, 17, 63 and 305.

Ala8. Number of clusters N_c = 8, core radius R_c = 0.10 nm

Table S12a.
Comparison of results from full K matrix (dimension N_c = 8) and reduced dimensionality models with dimensions N=2-5. Relaxation times, ns.

Full K matrix	N=2	N=3	N=4	N=5
12.7	12.6	12.6	12.6	12.7
3.2		3.2	3.2	3.2
2.6			2.6	2.6
2.5				2.4
2.0				

N=2
... Aggregate rates RHS

```
  1  -0.071492   0.007774
  2   0.071492  -0.007774
```

```
  #        NHB RMSD  Population     ΔG    Ns  Struct
  1   3.0    1.8  0.0980664    1.3227   3        helix
  2   0.1    3.9  0.9019336    0.0000   5        extended/PPII
```

...Aggregate distance matrix DA(I,J)

```
  1.8  4.0
  4.0  3.8
```

N=3
... Aggregate rates RHS

```
  1  -0.071581   0.020654   0.007545
  2   0.003461  -0.310272   0.005375
  3   0.068120   0.289618  -0.012920
```

```
  #        NHB RMSD  Population     ΔG    Ns  Struct
  1   3.0    1.8  0.0980664    1.3118   3        helix
  2   0.2    3.6  0.0164350    2.3766   1        turn
  3   0.1    4.0  0.8854986    0.0000   4        extended/PPII
```

...Aggregate distance matrix DA(I,J)

```
  1.8  3.9  4.0
  3.9  0.0  4.6
  4.0  4.6  3.3
```

N=4

1	-0.310275	0.006366	0.003455	0.005354
2	0.007546	-0.374647	0.003881	0.007845
3	0.020616	0.019539	-0.071658	0.007284
4	0.282113	0.348741	0.064321	-0.020483

#	NHB	RMSD	Population	ΔG	Ns	Struct
1	0.2	3.6	0.0164350	2.3633	1	turn
2	0.2	3.6	0.0194820	2.2620	1	turn
3	3.0	1.8	0.0980664	1.2985	3	helix
4	0.1	4.0	0.8660166	0.0000	3	extended/PPII

...Aggregate distance matrix DA(I,J)

0.0	3.4	3.9	5.0
3.4	0.0	3.3	3.9
3.9	3.3	1.8	4.3
5.0	3.9	4.3	2.7

N=5

... Aggregate rates RHS

1	-0.310308	0.006382	0.005793	0.002580	0.005341
2	0.007565	-0.374656	0.002496	0.004399	0.007853
3	0.010670	0.003879	-0.331124	0.093488	0.003967
4	0.010644	0.015311	0.209364	-0.146401	0.003596
5	0.281428	0.349084	0.113472	0.045933	-0.020756

#	NHB	RMSD	Population	ΔG	Ns	Struct
1	0.2	3.6	0.0164350	2.3633	1	turn
2	0.2	3.6	0.0194820	2.2620	1	turn
3	1.4	2.9	0.0302705	1.9993	2	nucleus*
4	3.7	1.3	0.0677959	1.5186	1	helix
5	0.1	4.0	0.8660166	0.0000	3	extended/PPII

*nucleus – 2...6 helical h-bond at center

...Aggregate distance matrix DA(I,J)

0.0	3.4	3.8	3.9	5.0
3.4	0.0	3.2	3.5	3.9
3.8	3.2	1.4	2.0	4.3
3.9	3.5	2.0	0.0	4.2
5.0	3.9	4.3	4.2	2.7

Ala8. Number of clusters N_c = 17, core radius R_c = 0.09 nm

Table S12b.

Comparison of results from full K matrix (dimension N_c = 17) and reduced dimensionality models with dimensions N=2-5. Relaxation times, ns.

Full K matrix	N=2	N=3	N=4	N=5
12.7	11.9	11.9	12.4	12.6
4.9		4.9	4.9	4.9
4.3			4.3	4.3
3.6				3.6
3.3				

N=2

1	-0.078730	0.005138
2	0.078730	-0.005138

#	NHB	RMSD	Population	ΔG	Ns	Struct
1	3.9	1.0	0.0612544	1.6271	2	helix
2	0.2	3.9	0.9387456	0.0000	15	extended/PPII

...Aggregate distance matrix DA(I,J)

1.4	3.4
3.4	3.6

N=3

... Aggregate rates RHS

1	-0.078731	-0.001767	0.005141
2	-0.000014	-0.204386	0.000107
3	0.078745	0.206152	-0.005249

#	NHB	RMSD	Population	ΔG	Ns	Struct
1	3.9	1.0	0.0612544	1.6268	2	helix
2	0.1	3.8	0.0004885	4.5070	1	beta turn*
3	0.2	3.9	0.9382571	0.0000	14	extended/PPII

...Aggregate distance matrix DA(I,J)

1.4	3.8	3.4
3.8	0.0	4.1
3.4	4.1	3.5

N=4

... Aggregate rates RHS

1	-0.084059	0.000427	0.039359	0.003205
2	0.000003	-0.204393	-0.000005	0.000114
3	0.038069	-0.000607	-0.212899	0.011698
4	0.045986	0.204573	0.173545	-0.015017

#	NHB	RMSD	Population	ΔG	Ns	Struct
1	3.9	1.0	0.0612544	1.5879	2	helix
2	0.1	3.8	0.0004885	4.4681	1	beta turn*

3	1.1	3.1	0.0592474	1.6078	3	turn
4	0.1	3.9	0.8790096	0.0000	11	extended/PPII

...Aggregate distance matrix DA(I,J)

1.4	3.8	3.0	3.5
3.8	0.0	3.7	4.2
3.0	3.7	2.7	3.6
3.5	4.2	3.6	3.5

N=5

... Aggregate rates RHS

1	-0.086685	0.000313	0.071000	0.015871	0.003448
2	0.000002	-0.204393	0.000006	-0.000013	0.000114
3	0.028145	0.000295	-0.238391	0.024940	0.003632
4	0.009060	-0.000940	0.035913	-0.245575	0.008146
5	0.049478	0.204725	0.131472	0.204777	-0.015340

#	NHB	RMSD	Population	ΔG	Ns	Struct
1	3.9	1.0	0.0612544	1.5879	2	helix
2	0.1	3.8	0.0004885	4.4681	1	beta turn*
3	1.5	2.8	0.0242815	2.1396	1	nucleus**
4	0.8	3.4	0.0349660	1.9222	2	turn
5	0.1	3.9	0.8790096	0.0000	11	extended/PPII

*beta turn has 3CO...HN6 and 3NH...OC6 bridging hydrogen bonds
*nucleus – 2CO...HN6 and 3CO...HN7 hydrogen bonds

...Aggregate distance matrix DA(I,J)

1.4	3.8	2.4	3.3	3.5
3.8	0.0	4.1	3.5	4.2
2.4	4.1	0.0	2.8	3.5
3.3	3.5	2.8	2.5	3.7
3.5	4.2	3.5	3.7	3.5

Ala8. Number of clusters N_c = 63, core radius R_c = 0.07 nm

Table S12c.
Comparison of results from full K matrix (dimension N_c =63) and reduced dimensionality models with dimensions N=2-5. Relaxation times, ns.

Full K matrix	N=2	N=3	N=4	N=5
13.7	13.4	13.5	13.5	13.5
5.4		5.4	5.4	5.4
5.1			5.1	5.1
4.0				3.8

3.5				

N=2
... Aggregate rates RHS

1	-0.070128	0.004174
2	0.070128	-0.004174

#	NHB	RMSD	Population	ΔG	Ns	Struct
1	4.4	0.8	0.0561799	1.6819	3	helix
2	0.1	3.9	0.9438201	0.0000	60	extended

...Aggregate distance matrix DA(I,J)

1.7	3.5
3.5	3.4

N=3
... Aggregate rates RHS

1	-0.070130	-0.000785	0.004177
2	-0.000007	-0.186282	0.000106
3	0.070137	0.187067	-0.004283

#	NHB	RMSD	Population	ΔG	Ns	Struct
1	4.4	0.8	0.0561799	1.6816	3	helix
2	0.9	3.5	0.0005325	4.4588	1	turn
3	0.1	3.9	0.9432876	0.0000	59	extended

...Aggregate distance matrix DA(I,J)

1.7	3.6	3.5
3.6	0.0	3.6
3.5	3.6	3.4

N=4

1	-0.195515	-0.000173	0.000234	0.000405
2	-0.000046	-0.186283	-0.000007	0.000106
3	0.006509	-0.000778	-0.070131	0.004172
4	0.189051	0.187234	0.069904	-0.004683

#	NHB	RMSD	Population	ΔG	Ns	Struct
1	1.4	3.8	0.0020155	3.6640	2	turn
2	0.9	3.5	0.0005325	4.4575	1	turn
3	4.4	0.8	0.0561799	1.6803	3	helix
4	0.1	3.9	0.9412721	0.0000	57	extended

...Aggregate distance matrix DA(I,J)

1.6	2.6	3.8	3.8
2.6	0.0	3.6	3.7
3.8	3.6	1.7	3.5

3.8   3.7   3.5   3.3

N=5
... Aggregate rates RHS

1	-0.252770	0.006713	-0.001028	0.008137	0.004651
2	0.000190	-0.186284	-0.000044	-0.000010	0.000104
3	-0.000110	-0.000167	-0.195525	0.000247	0.000415
4	0.024331	-0.001016	0.006892	-0.070668	0.003794
5	0.228358	0.180754	0.189704	0.062294	-0.008963

#	NHB	RMSD	Population	ΔG	Ns	Struct
1	1.5	3.2	0.0187850	2.3213	2	turn
2	0.9	3.5	0.0005325	4.4455	1	turn
3	1.4	3.8	0.0020155	3.6520	2	turn
4	4.4	0.8	0.0561799	1.6683	3	helix
5	0.1	3.9	0.9224871	0.0000	55	extended

...Aggregate distance matrix DA(I,J)

2.0	2.9	3.0	3.3	3.4
2.9	0.0	2.6	3.6	3.7
3.0	2.6	1.6	3.8	3.8
3.3	3.6	3.8	1.7	3.5
3.4	3.7	3.8	3.5	3.3

Ala8. Number of clusters N_c = 305, core radius R_c = 0.06 nm

Table S12d.
Comparison of results from full K matrix (dimension N_c =63) and reduced dimensionality models with dimensions N=2-5. Relaxation times, ns.

Full K matrix	N=2	N=3	N=4	N=5
13.4	12.6	12.6	12.6	12.6
5.8		5.8	5.8	5.8
3.8			3.8	3.8
3.1				3.0
3.0				

N=2
... Aggregate rates RHS

1	-0.075756	0.003637
2	0.075756	-0.003637

#	NHB	RMSD	Population	ΔG	Ns	Struct
1	4.8	0.5	0.0458120	1.8100	7	helix
2	0.2	3.9	0.9541882	0.0000	298	extended

N=3

... Aggregate rates RHS

1	-0.075758	0.006594	0.003632
2	0.000240	-0.172402	0.000291
3	0.075517	0.165808	-0.003922

#	NHB	RMSD	Population	ΔG	Ns	Struct
1	4.8	0.5	0.0458120	1.8090	7	helix
2	1.5	3.8	0.0016695	3.7834	2	turn
3	0.2	3.9	0.9525187	0.0000	296	extended

N=4

... Aggregate rates RHS

1	-0.172402	0.000163	0.000240	0.000291
2	0.000018	-0.261832	-0.000005	0.000052
3	0.006594	-0.001280	-0.075758	0.003633
4	0.165790	0.262949	0.075523	-0.003975

#	NHB	RMSD	Population	ΔG	Ns	Struct
1	1.5	3.8	0.0016695	3.7833	2	turn
2	1.1	3.2	0.0001880	5.0851	2	turn
3	4.8	0.5	0.0458120	1.8089	7	helix
4	0.2	3.9	0.9523307	0.0000	294	extended

N=5

... Aggregate rates RHS

1	-0.336457	-0.000028	0.000009	0.000044	0.000043
2	-0.000041	-0.261832	0.000018	-0.000005	0.000052
3	0.000121	0.000163	-0.172402	0.000240	0.000291
4	0.016077	-0.001261	0.006614	-0.075750	0.003643
5	0.320299	0.262958	0.165760	0.075470	-0.004028

#	NHB	RMSD	Population	ΔG	Ns	Struct
1	0.9	3.8	0.0001265	5.3211	2	turn
2	1.1	3.2	0.0001880	5.0849	2	turn
3	1.5	3.8	0.0016695	3.7831	2	turn
4	4.8	0.5	0.0459560	1.8068	8	helix
5	0.2	3.9	0.9520602	0.0000	291	extended

Ala_{15} ODR results

2 x 10 μs MD trajectories at 300 K
Discretization by clustering with CA atom RMSD for number of clusters N_c = 11, 45, 134 and 491.

Ala15. Number of clusters N_c = 11, core radius R_c = 0.25 nm

Table S13a.
Comparison of results from full K matrix (dimension N_c = 11) and reduced dimensionality models with dimensions N=2-5. Relaxation times, ns.

Full K matrix	N=2	N=3	N=4	N=5
106.	105.	106.	106.	106.
17.8		17.8	17.8	17.8
6.3			5.9	6.0
5.2				5.1
5.0				

N=2
... Aggregate rates RHS

```
 1  -0.006662   0.002900
 2   0.006662  -0.002900
```

```
 #        NHB RMSD  Population      ΔG     Ns  Struct
 1    9.2    2.2  0.3029442    0.4968   1     N-term helix
 2    0.6    5.9  0.6970558    0.0000   10    central helix
```

...Aggregate distance matrix DA(I,J)

```
 0.0   6.2
 6.2   7.2
```

N=3
... Aggregate rates RHS

```
 1  -0.007093   0.016520   0.002315
 2   0.002064  -0.054048   0.002158
 3   0.005029   0.037528  -0.004473
```

```
 #        NHB RMSD  Population      ΔG     Ns  Struct
 1    9.2    2.2  0.3029442    0.4635   1     N-term helix
 2    4.7    4.6  0.0378475    1.7034   1     central helix
 3    0.4    6.0  0.6592083    0.0000   9     extended
```

...Aggregate distance matrix DA(I,J)

```
 0.0   6.0   6.2
```

6.0	0.0	6.3
6.2	6.3	7.4

... Aggregate rates RHS

1	-0.054079	0.005881	0.002066	0.002080
2	0.002120	-0.165613	0.000003	0.003381
3	0.016539	0.000068	-0.007094	0.002362
4	0.035419	0.159663	0.005025	-0.007823

#	NHB	RMSD	Population	ΔG	Ns	Struct
1	4.7	4.6	0.0378475	1.6910	1	central helix
2	0.5	6.4	0.0136465	2.2991	1	turn
3	9.2	2.2	0.3029442	0.4510	1	N-term helix
4	0.4	6.0	0.6455619	0.0000	8	extended

...Aggregate distance matrix DA(I,J)

0.0	5.0	6.0	6.5
5.0	0.0	7.0	7.5
6.0	7.0	0.0	6.1
6.5	7.5	6.1	7.4

N=5

... Aggregate rates RHS

1	-0.054097	0.004420	0.005766	0.002064	0.001998
2	0.002702	-0.186349	0.014857	0.000327	0.006289
3	0.002079	0.008763	-0.165877	-0.000001	0.003191
4	0.016524	0.004283	-0.000026	-0.007095	0.002294
5	0.032791	0.168882	0.145279	0.004705	-0.013772

#	NHB	RMSD	Population	ΔG	Ns	Struct
1	4.7	4.6	0.0378475	1.6692	1	central helix
2	0.8	5.9	0.0231370	1.9626	2	turn
3	0.5	6.4	0.0136465	2.2773	1	turn
4	9.2	2.2	0.3029442	0.4293	1	N-term helix
5	0.4	6.0	0.6224249	0.0000	6	extended

...Aggregate distance matrix DA(I,J)

0.0	4.8	5.0	6.0	7.0
4.8	5.5	5.2	6.8	7.9
5.0	5.2	0.0	7.0	8.3
6.0	6.8	7.0	0.0	5.8
7.0	7.9	8.3	5.8	7.1

Ala15. Number of clusters N_c = 45, core radius R_c = 0.225 nm

Table S13b.

Comparison of results from full K matrix (dimension N_c = 45) and reduced dimensionality models with dimensions N=2-5. Relaxation times, ns.

Full K matrix	N=2	N=3	N=4	N=5
109.	103.	106.	107.	107.0
17.6		17.1	17.3	17.3
15.7			15.3	15.3
11.9				11.8
8.9				

N=2

... Aggregate rates RHS

1	-0.006486	0.003223
2	0.006486	-0.003223

#	NHB	RMSD	Population	ΔG	Ns	Struct
1	8.9	2.4	0.3319562	0.4169	5	helix
2	0.4	6.0	0.6680439	0.0000	40	extended

...Aggregate distance matrix DA(I,J)

4.9	5.7
5.7	6.3

N=3

... Aggregate rates RHS

1	-0.010135	0.029817	0.001700
2	0.006022	-0.053949	0.002015
3	0.004112	0.024132	-0.003715

#	NHB	RMSD	Population	ΔG	Ns	Struct
1	9.5	2.0	0.2761752	0.5266	3	helix
2	5.8	4.2	0.0557809	1.4801	2	C-term helix
3	0.4	6.0	0.6680439	0.0000	40	extended

...Aggregate distance matrix DA(I,J)

4.4	5.0	5.8
5.0	6.1	5.7
5.8	5.7	6.3

N=4

... Aggregate rates RHS

1	-0.062543	0.000345	0.000665	0.002728
2	0.001027	-0.054089	0.002045	0.006280
3	0.023547	0.024355	-0.003921	0.003083
4	0.037970	0.029389	0.001211	-0.012090

#	NHB	RMSD	Population	ΔG	Ns	Struct
1	5.6	4.6	0.0187540	2.1267	2	N-term helix

2	5.8	4.2	0.0557809	1.4769	2	C-term helix
3	0.4	6.0	0.6644129	0.0000	39	extended
4	9.6	1.9	0.2610522	0.5569	2	helix

...Aggregate distance matrix DA(I,J)

4.0	5.2	5.8	5.5
5.2	6.1	5.7	5.0
5.8	5.7	6.3	5.9
5.5	5.0	5.9	3.9

N=5

... Aggregate rates RHS

1	-0.054114	0.006165	-0.000135	0.002035	0.006306
2	0.000401	-0.081737	0.003638	0.000266	0.000163
3	-0.000037	0.015154	-0.065268	0.000407	0.002543
4	0.024234	0.048674	0.017864	-0.003970	0.003215
5	0.029515	0.011744	0.043901	0.001263	-0.012228

#	NHB	RMSD	Population	ΔG	Ns	Struct
1	5.8	4.2	0.0557809	1.4769	2	C-term helix
2	2.7	5.3	0.0036310	3.1055	1	turn/central helix
3	6.3	4.4	0.0151230	2.2550	1	N-term helix
4	0.4	6.0	0.6644129	0.0000	39	extended
5	9.6	1.9	0.2610522	0.5569	2	helix

...Aggregate distance matrix DA(I,J)

6.1	5.4	4.9	5.7	5.0
5.4	0.0	4.0	5.9	6.3
4.9	4.0	0.0	5.6	4.7
5.7	5.9	5.6	6.3	5.9
5.0	6.3	4.7	5.9	3.9

Ala15. Number of clusters N_c = 134, core radius R_c = 0.20 nm

Table S13c.
Comparison of results from full K matrix (dimension N_c = 134) and reduced dimensionality models with dimensions N=2-5. Relaxation times, ns.

Full K matrix	N=2	N=3	N=4	N=5
107.	101.	103.	102.	102.0
24.3		23.1	23.1	23.5
18.9			18.1	18.3
13.7				12.6
12.6				

N=2

... Aggregate rates RHS

1	-0.006727	0.003214
2	0.006727	-0.003214

#	NHB	RMSD	Population	ΔG	Ns	Struct
1	9.0	2.3	0.3232912	0.4404	17	helix
2	0.5	6.0	0.6767089	0.0000	117	extended

...Aggregate distance matrix DA(I,J)

4.5	5.5
5.5	5.9

N=3

... Aggregate rates RHS

1	-0.040056	0.001332	0.004303
2	0.017443	-0.003439	0.005248
3	0.022614	0.002107	-0.009551

#	NHB	RMSD	Population	ΔG	Ns	Struct
1	6.0	4.2	0.0516765	1.5334	5	C-term helix
2	0.5	6.0	0.6767089	0.0000	117	extended
3	9.6	1.9	0.2716147	0.5442	12	helix

...Aggregate distance matrix DA(I,J)

4.1	5.4	4.6
5.4	5.9	5.5
4.6	5.5	4.5

N=4

... Aggregate rates RHS

1	-0.039991	-0.000288	0.004171	0.001292
2	-0.000005	-0.054773	-0.000003	0.000071
3	0.023629	-0.001084	-0.009409	0.002343
4	0.016366	0.056145	0.005241	-0.003706

#	NHB	RMSD	Population	ΔG	Ns	Struct
1	6.0	4.2	0.0516765	1.5137	5	C-term helix
2	1.3	6.1	0.0008285	3.9776	1	turn
3	9.2	2.1	0.2927347	0.4799	15	helix
4	0.4	6.0	0.6547605	0.0000	113	extended

...Aggregate distance matrix DA(I,J)

4.1	5.7	4.7	5.4
5.7	0.0	6.2	6.7
4.7	6.2	4.9	5.6
5.4	6.7	5.6	5.9

N=5

... Aggregate rates RHS

1	-0.063331	-0.000037	0.022326	0.003485	0.000547
2	-0.000001	-0.054773	-0.000009	-0.000003	0.000071
3	0.016218	-0.000223	-0.054866	0.000819	0.000692
4	0.034910	-0.001125	0.011294	-0.009679	0.002414
5	0.012205	0.056157	0.021254	0.005379	-0.003723

#	NHB	RMSD	Population	ΔG	Ns	Struct
1	6.4	4.1	0.0293245	1.8515	3	helix-turn-helix
2	1.3	6.1	0.0008285	3.9776	1	turn
3	5.7	4.4	0.0213015	2.0420	1	C-term helix
4	9.2	2.1	0.2937852	0.4777	16	helix
5	0.4	6.0	0.6547605	0.0000	113	extended

...Aggregate distance matrix DA(I,J)

4.4	5.6	3.7	4.6	5.3
5.6	0.0	5.7	6.2	6.7
3.7	5.7	0.0	4.8	5.2
4.6	6.2	4.8	4.9	5.6
5.3	6.7	5.2	5.6	5.9

Ala15. Number of clusters N_c = 491, core radius R_c = 0.187nm

Table S13d.
Comparison of results from full K matrix (dimension N_c = 134) and reduced dimensionality models with dimensions N=2-5. Relaxation times, ns.

Full K matrix	N=2	N=3	N=4	N=5
76.	71.	72.	72.	72.0
30.8		30.1	30.1	30.1
20.4			20.3	20.3
11.6				8.3
9.9				

N=2
... Aggregate rates RHS

1	-0.009221	0.004775
2	0.009221	-0.004775

#	NHB	RMSD	Population	ΔG	Ns	Struct
1	8.8	2.4	0.3410327	0.3927	55	helix
2	0.4	6.0	0.6589676	0.0000	436	extended

N=3
... Aggregate rates RHS

1	-0.009387	-0.000950	0.004589
2	-0.000003	-0.033196	0.000047
3	0.009390	0.034146	-0.004636

#	NHB	RMSD	Population	ΔG	Ns	Struct
1	9.0	2.3	0.3278347	0.4272	42	helix
2	1.4	5.8	0.0009170	3.9319	4	turn
3	0.4	6.0	0.6712486	0.0000	445	extended

N=4

71.650415

... Aggregate rates RHS

1	-0.049241	-0.000024	0.000104	0.000095
2	-0.000011	-0.033196	-0.000003	0.000047
3	0.017236	-0.000918	-0.009407	0.004561
4	0.032017	0.034139	0.009306	-0.004703

#	NHB	RMSD	Population	ΔG	Ns	Struct
1	4.0	5.6	0.0019795	3.4715	2	N-term helix
2	1.4	5.8	0.0009170	3.9302	4	turn
3	9.0	2.3	0.3278347	0.4254	42	helix
4	0.4	6.0	0.6692691	0.0000	443	extended

N=5

... Aggregate rates RHS

1	-0.049250	-0.000028	0.000106	-0.000048	0.000123
2	-0.000013	-0.033197	0.000049	0.000003	-0.000002
3	0.033613	0.033485	-0.009340	0.062190	0.001451
4	-0.002086	0.000266	0.008525	-0.109106	0.014166
5	0.017735	-0.000527	0.000659	0.046961	-0.015738

#	NHB	RMSD	Population	ΔG	Ns	Struct
1	4.0	5.6	0.0019795	3.4323	2	N-term helix
2	1.4	5.8	0.0009170	3.8910	4	turn
3	0.3	6.0	0.6266721	0.0000	381	extended
4	3.8	4.8	0.0858465	1.1850	82	C-term helix
5	9.5	2.0	0.2845852	0.4706	22	helix

Ala_{21} ODR results

Ala21. Number of clusters N_c = 34, core radius R_c = 0.25 nm

Table S14a.
Comparison of results from full K matrix (dimension N_c = 34) and reduced dimensionality models with dimensions N=2-5. Relaxation times, ns.

Full K matrix	N=2	N=3	N=4	N=5
400.	374	376	379	383
238		237	238	238
119			108	111
112				105
86				

N=2
... Aggregate rates RHS

1	-0.000540	0.002131
2	0.000540	-0.002131

#	NHB	RMSD	Population	ΔG	Ns	Struct
1	13.6	3.0	0.7976521	0.0000	7	helix
2	1.7	7.5	0.2023479	0.8177	27	extened

...Aggregate distance matrix DA(I,J)

7.6	8.2
8.2	9.0

N=3
... Aggregate rates RHS

1	-0.004090	0.000083	0.000010
2	0.002708	-0.002242	0.000530
3	0.001382	0.002158	-0.000541

#	NHB	RMSD	Population	ΔG	Ns	Struct
1	2.8	7.4	0.0060193	2.9131	1	turn
2	1.7	7.5	0.1963287	0.8357	26	extended
3	13.6	3.0	0.7976521	0.0000	7	helix

...Aggregate distance matrix DA(I,J)

0.0	8.9	8.5
8.9	9.0	8.2
8.5	8.2	7.6

N=4
... Aggregate rates RHS

1	-0.009044	0.000064	0.000315	0.000094
2	0.000025	-0.004090	0.000082	0.000010
3	0.004215	0.002764	-0.002357	0.000515
4	0.004803	0.001262	0.001960	-0.000619

#	NHB	RMSD	Population	ΔG	Ns	Struct
1	6.1	6.6	0.0152037	2.3435	3	helix-turn-helix
2	2.8	7.4	0.0060193	2.8958	1	turn
3	1.9	7.4	0.2038852	0.7959	27	extended
4	13.8	2.9	0.7748919	0.0000	3	helix

...Aggregate distance matrix DA(I,J)

7.7	7.6	8.2	7.2
7.6	0.0	9.1	8.1
8.2	9.1	9.0	8.0
7.2	8.1	8.0	7.4

N=5

... Aggregate rates RHS

1	-0.004091	0.000055	0.000022	0.000084	0.000010
2	0.000144	-0.009154	0.000007	0.000383	0.000092
3	0.000048	0.000006	-0.008908	0.000261	0.000090
4	0.002602	0.004571	0.003647	-0.002545	0.000438
5	0.001297	0.004522	0.005231	0.001817	-0.000630

#	NHB	RMSD	Population	ΔG	Ns	Struct
1	2.8	7.4	0.0060193	2.8975	1	turn
2	3.8	6.6	0.0157285	2.3249	2	central helix
3	6.6	6.5	0.0133970	2.4206	2	helix-turn-helix
4	1.7	7.5	0.1877984	0.8466	24	extended
5	13.8	2.9	0.7770569	0.0000	5	helix

...Aggregate distance matrix DA(I,J)

0.0	8.7	8.3	8.9	8.7
8.7	9.7	8.0	8.4	8.1
8.3	8.0	7.2	7.9	6.8
8.9	8.4	7.9	9.1	8.4
8.7	8.1	6.8	8.4	8.1

Ala21. Number of clusters N_c = 76, core radius R_c = 0.24 nm

Table S14b.

Comparison of results from full K matrix (dimension N_c = 76) and reduced dimensionality models with dimensions N=2-5. Relaxation times, ns.

Full K matrix	N=2	N=3	N=4	N=5

456.	441	442	440	443
221		196	196	196
130			105	108
98				79
94				

N=2

... Aggregate rates RHS

```
  1  -0.000541   0.001725
  2   0.000541  -0.001725
```

#	NHB	RMSD	Population	ΔG	Ns	Struct
1	13.9	2.8	0.7608119	0.0000	12	helix
2	2.4	7.4	0.2391882	0.6898	64	extended

...Aggregate distance matrix DA(I,J)

```
  6.6   7.8
  7.8   8.5
```

N=3

... Aggregate rates RHS

```
  1  -0.004977   0.000135   0.000028
  2   0.002964  -0.001848   0.000514
  3   0.002013   0.001712  -0.000541
```

#	NHB	RMSD	Population	ΔG	Ns	Struct
1	8.0	7.2	0.0104063	2.5586	3	helix-turn-helix
2	2.1	7.4	0.2287819	0.7163	61	extended
3	13.9	2.8	0.7608119	0.0000	12	helix

...Aggregate distance matrix DA(I,J)

```
  6.8   7.8   6.7
  7.8   8.6   7.8
  6.7   7.8   6.6
```

N=4

... Aggregate rates RHS

```
  1  -0.004978   0.000009   0.000139   0.000028
  2   0.000004  -0.009409   0.000160   0.000015
  3   0.002949   0.007104  -0.002009   0.000496
  4   0.002025   0.002296   0.001711  -0.000539
```

#	NHB	RMSD	Population	ΔG	Ns	Struct
1	8.0	7.2	0.0104063	2.5602	3	helix-turn-helix
2	1.8	7.5	0.0049737	3.0003	3	turn
3	2.1	7.4	0.2217444	0.7366	57	extended

4 13.9 2.8 0.7628756 0.0000 13 helix

...Aggregate distance matrix DA(I,J)

6.8	7.8	7.9	6.7
7.8	10.2	8.7	7.7
7.9	8.7	8.6	7.8
6.7	7.7	7.8	6.7

N=5

... Aggregate rates RHS

1	-0.004982	0.000033	-0.000044	0.000147	0.000028
2	0.000016	-0.009484	0.000745	0.000132	0.000013
3	-0.000055	0.001945	-0.012058	0.000437	0.000074
4	0.002948	0.005555	0.007021	-0.002304	0.000435
5	0.002072	0.001951	0.004336	0.001588	-0.000550

#	NHB	RMSD	Population	ΔG	Ns	Struct
1	8.0	7.2	0.0104063	2.5597	3	helix-turn-helix
2	1.8	7.5	0.0049737	2.9998	3	turn
3	4.9	6.7	0.0129902	2.4275	9	central helix
4	1.9	7.4	0.2093417	0.7704	50	extended
5	13.9	2.8	0.7622881	0.0000	11	helix

...Aggregate distance matrix DA(I,J)

6.8	7.8	7.1	7.9	6.7
7.8	10.2	8.0	8.8	7.7
7.1	8.0	7.8	8.2	7.2
7.9	8.8	8.2	8.7	7.9
6.7	7.7	7.2	7.9	6.7

Ala21. Number of clusters N_c = 194, core radius R_c = 0.22 nm

Table S14c.
Comparison of results from full K matrix (dimension N_c = 194) and reduced dimensionality models with dimensions N=2-5. Relaxation times, ns.

Full K matrix	N=2	N=3	N=4	N=5
377	289	340	351	350
281		250	259	256
202			185	185
145				125
139				

N=2

... Aggregate rates RHS

1	-0.003441	0.000013
2	0.003441	-0.000013

#	NHB	RMSD	Population	ΔG	Ns	Struct
1	1.0	7.6	0.0035362	3.3628	6	N-term helix nucleus
2	11.2	3.9	0.9964639	0.0000	188	helix

N=3

... Aggregate rates RHS

1	-0.002525	0.004766	0.000661
2	0.000059	-0.003737	-0.000003
3	0.002466	-0.001029	-0.000657

#	NHB	RMSD	Population	ΔG	Ns	Struct
1	0.8	7.6	0.0048997	3.1640	8	N-term helix nucleus
2	2.4	7.5	0.0060008	3.0432	9	turn
3	11.3	3.8	0.9890996	0.0000	177	helix

N=4

... Aggregate rates RHS

1	-0.005379	-0.000007	0.000036	0.000016
2	-0.000006	-0.003515	0.000071	-0.000002
3	0.001801	0.003995	-0.002621	0.000574
4	0.003584	-0.000473	0.002513	-0.000588

#	NHB	RMSD	Population	ΔG	Ns	Struct
1	1.0	7.6	0.0035362	3.2288	6	N-term helix nucleus
2	3.6	7.4	0.0024385	3.4504	5	central helix
3	1.7	7.5	0.1982267	0.8286	149	extended
4	13.6	2.9	0.7957986	0.0000	34	helix

N=5

... Aggregate rates RHS

1	-0.005380	-0.000001	-0.000005	0.000038	0.000016
2	-0.000000	-0.007899	0.000008	0.000073	0.000006
3	-0.000005	0.000014	-0.003524	0.000085	-0.000001
4	0.001721	0.005544	0.003737	-0.002796	0.000533
5	0.003664	0.002342	-0.000216	0.002600	-0.000555

#	NHB	RMSD	Population	ΔG	Ns	Struct
1	11.4	7.7	0.0036870	3.2266	2	helix-turn-helix
2	1.0	7.6	0.0035362	3.2515	6	N-term helix nucleus
3	3.0	7.7	0.0031430	3.3217	6	central helix
4	1.0	7.6	0.1630022	0.9679	129	extended
5	13.2	3.1	0.8266316	0.0000	51	helix

Ala21. Number of clusters N_c = 605, core radius R_c = 0.24 nm

Table S14d.
Comparison of results from full K matrix (dimension N_c = 605) and reduced dimensionality models with dimensions N=2-5. Relaxation times, ns.

Full K matrix	N=2	N=3	N=4	N=5
303	272	273	282	283
240		80	218	218
112			100	100
82				74
66				

N=2
... Aggregate rates RHS

1	-0.000006	0.003675
2	0.000006	-0.003675

#	NHB	RMSD	Population	ΔG	Ns	Struct
1	11.2	3.9	0.9984139	0.0000	596	helix
2	1.6	8.4	0.0015862	3.8419	9	central helix

N=3
... Aggregate rates RHS

1	-0.003686	0.000109	0.000003
2	0.002101	-0.012101	0.000442
3	0.001585	0.011992	-0.000445

#	NHB	RMSD	Population	ΔG	Ns	Struct
1	1.5	8.2	0.0018450	3.7301	11	central helix
2	0.7	7.8	0.0354427	1.9683	188	extended
3	11.5	3.8	0.9627124	0.0000	406	helix

N=4
... Aggregate rates RHS

1	-0.003674	-0.000026	0.000041	-0.000001
2	-0.000018	-0.009835	0.000079	-0.000002
3	0.004014	0.011320	-0.003830	0.000752
4	-0.000322	-0.001459	0.003710	-0.000749

#	NHB	RMSD	Population	ΔG	Ns	Struct
1	1.4	8.3	0.0017022	3.6891	10	central helix
2	1.9	6.9	0.0011712	3.9119	8	turn
3	0.7	7.7	0.1680562	0.9514	424	extended
4	13.3	3.1	0.8290704	0.0000	163	helix

N=5

... Aggregate rates RHS

1	-0.003674	-0.000024	-0.000026	0.000041	-0.000001
2	-0.000015	-0.013304	0.000019	0.000090	-0.000001
3	-0.000018	0.000020	-0.009835	0.000080	-0.000002
4	0.004038	0.013940	0.011313	-0.003971	0.000753
5	-0.000332	-0.000633	-0.001470	0.003760	-0.000749

#	NHB	RMSD	Population	ΔG	Ns	Struct
1	1.4	8.3	0.0017022	3.6896	10	central helix
2	1.1	6.8	0.0010692	3.9668	7	turn
3	1.9	6.9	0.0011712	3.9125	8	turn
4	0.7	7.8	0.1661550	0.9588	412	extended
5	13.3	3.1	0.8299024	0.0000	168	alpha

REFERENCES

1. Jas, G. S.; Kuczera, K., Helix-Coil Transition Courses Through Multiple Pathways and Intermediates: Fast Kinetic Measurements and Dimensionality Reduction. *J Phys Chem B* **2018,** *122* (48), 10806-10816.
2. Buchete, N. V.; Hummer, G., Coarse master equations for peptide folding dynamics. *J Phys Chem B* **2008,** *112* (19), 6057-6069.
3. Kube, S.; Weber, M., A coarse graining method for the identification of transition rates between molecular conformations. *J Chem Phys* **2007,** *126* (2).
4. Senne, M.; Trendelkamp-Schroer, B.; Mey, A. S. J. S.; Schutte, C.; Noe, F., EMMA: A Software Package for Markov Model Building and Analysis. *J Chem Theory Comput* **2012,** *8* (7), 2223-2238.
5. Hummer, G.; Szabo, A., Optimal Dimensionality Reduction of Multistate Kinetic and Markov-State Models. *J Phys Chem B* **2015,** *119* (29), 9029-37.
